# Unsupervised Machine Learning for the Identification of Latent First-Trimester Obstetric Phenotypes Associated with Maternal and Perinatal Morbidity

**DOI:** 10.3390/diagnostics16162654

**Published:** 2026-08-20

**Authors:** Alexandra-Elena Cristofor, Alexandru Carauleanu, Ingrid-Andrada Vasilache, Iustina Condriuc, Ioana Rosu, Carolina Susanu, Dragos Nemescu

**Affiliations:** 1 Grigore T. Popa University of Medicine and Pharmacy, 700115 Iasi, Romaniaioanabojescu@gmail.com (I.R.);; 2 Faculty of Medicine and Biological Sciences, ‘Ștefan cel Mare’ University, 720229 Suceava, Romania; tanasaingrid@yahoo.com; 3 Faculty of Medicine and Pharmacy, “Dunărea de Jos” University, 47 Domnească Street, 800008 Galati, Romania

**Keywords:** unsupervised learning, phenotype clustering, pregnancy complications

## Abstract

**Background/Objectives**: Artificial intelligence may improve obstetric risk stratification by identifying clinically meaningful patterns that are not captured by single-outcome prediction models. This study evaluated whether unsupervised machine learning applied to first-trimester maternal, biophysical, and biochemical data could identify phenotypes associated with adverse obstetric outcomes. **Methods**: We analyzed 1583 first-trimester records that were ambispectively collected including maternal characteristics, obstetric history, mean arterial pressure, uterine artery pulsatility index, nuchal translucency, and PAPP-A. Principal component analysis followed by k-means clustering was used to derive AI phenotypes. Associations were tested for hypertensive, metabolic, delivery, neonatal, and actionable obstetric outcomes. Robustness of the clustering solution and phenotype–outcome associations was assessed using inverse probability weighting, false discovery rate correction, adverse-event burden scoring, cross-algorithm agreement analyses, and multiple sensitivity analyses. **Results**: Four clinically interpretable phenotypes were identified. Phenotype 4 represented a cardiometabolic–vascular subgroup that showed the greatest enrichment for adverse obstetric outcomes. It had higher rates of maternal metabolic or hypertensive complications (52.4%; RR 4.31) and clinically actionable adverse pregnancy outcomes (57.1%; RR 2.31). These associations remained significant after false discovery rate correction and were consistent in inverse probability weighting analyses. Phenotype 4 also had the highest cumulative adverse-event burden score (1.29 versus 0.35–0.47). Exploratory analyses demonstrated high specificity (97.2%) but limited sensitivity for clinically actionable adverse pregnancy outcomes. Incremental discrimination over conventional predictors was observed only for selected secondary outcomes, including early preterm birth and low Apgar score. **Conclusions**: Unsupervised AI phenotyping identified a cardiometabolic–vascular pregnancy subgroup associated with increased obstetric morbidity.

## 1. Introduction

Hypertensive disorders of pregnancy, gestational diabetes, spontaneous preterm birth, and disorders of fetal growth remain the dominant contributors to maternal and perinatal morbidity worldwide, and their consequences extend beyond delivery so that patients who experience adverse pregnancy outcomes carry an elevated lifetime risk of cardiovascular and metabolic disease, and their offspring inherit a comparable long-term liability [1,2].

First-trimester screening has evolved in parallel with this recognition, from a purely aneuploidy-focused examination into an integrated prognostic assessment combining maternal history, mean arterial pressure, uterine artery Doppler, and placental biomarkers [3]. The Fetal Medicine Foundation competing-risks model combines maternal factors with uterine artery pulsatility index, mean arterial pressure, and placental growth factor to detect approximately 75% of preterm preeclampsia at a 10% false-positive rate, a performance repeatedly reproduced in derivation and external validation cohorts [4,5,6]. Prospective validation in unselected nulliparous women has confirmed detection rates of 63–77% for preterm and early-onset preeclampsia, and pragmatic screen-and-prevent programmes using this model have redefined the standard of care [7,8].

First-trimester prediction models have been developed independently for preeclampsia, small-for-gestational-age birth, spontaneous preterm birth, and gestational diabetes, and the systematic evidence indicates that a patient flagged as low risk by any single model may still be carrying a clinically important constellation of risk factors that the current outcome-specific paradigm does not integrate [9,10]. Also, adverse pregnancy outcomes are strongly interrelated and cardiometabolic risk factors, such as obesity, insulin resistance, chronic hypertension, dyslipidaemia, cluster within the same pregnancies and drive both hypertensive disorders and gestational diabetes through overlapping vascular and metabolic pathways [11,12,13,14].

Literature data has shown that the co-occurrence of metabolic abnormalities in early pregnancy and their clustering with advancing maternal age carries a dose–response relationship with the composite risk of gestational hypertension, gestational diabetes, primary caesarean section, and preterm birth [15,16]. Chronic hypertension was associated with a five-fold increase in preeclampsia (adjusted OR 5.43, 95% CI 3.85–7.65), a two-fold increase in preterm birth, and comparable increases in caesarean delivery and small-for-gestational-age birth, and this excess risk was only partially attenuated by aspirin prophylaxis [17]. Thus, patients who enter pregnancy with several such risk factors are the archetypal candidates for integrated, rather than condition-specific, surveillance.

Unsupervised machine learning offers a natural framework for identifying these globally high-risk pregnancies [18,19]. Rather than optimising a classifier for a pre-specified label, clustering methods derive latent groupings directly from the joint structure of the input variables, allowing internally coherent clustering solution subgroups to emerge unsupervised [20].

Applied to preeclampsia, this approach has repeatedly recovered clinically meaningful subphenotypes: k-means and UMAP-based clustering of maternal and angiogenic variables identified three preeclampsia phenotypes with distinct rates of maternal and neonatal complications [21]. The k-means clustering of 26 routinely collected variables in >2300 women defined four phenotypes with a relative risk of 2.83 (95% CI 2.48–3.23) for adverse perinatal outcomes in the highest-risk group [22]. Also, an organ-system-based clustering in more than 7500 PE patients demonstrated a cross-validated stability with an adjusted Rand index near 0.88 [23].

Similar approaches have been applied to gestational diabetes, identifying four data-driven clusters with differential risks of perinatal and postpartum outcomes, and to placental histopathology and molecular data, where they have consistently mapped onto clinically distinct trajectories [24,25].

Only a small fraction of the pregnancy machine-learning literature is unsupervised, 9 of 127 studies in the most recent systematic review, and most existing work has been directed at outcome-specific supervised prediction rather than at phenotype discovery [26].

In this study we therefore performed an unsupervised phenotyping analysis of 1583 singleton pregnancies undergoing routine first-trimester screening, using only maternal, biophysical, and biochemical variables available at 11–13 weeks. Our objective was to determine whether an interpretable high-risk phenotype could be contoured from routine first-trimester data, and whether it could serve as a complementary risk-enrichment tool alongside existing outcome-specific algorithms.

## 2. Materials and Methods

### 2.1. Study Design and Population

This ambispective observational cohort study included 1583 singleton pregnancies of Caucasian ethnicity undergoing routine first-trimester screening in a private practice clinic from Iasi, Romania, between January 2012–July 2026. The retrospective component comprised 1452 singleton pregnancies recorded between 2012 and 2021 in Astraia (version 30.1.2, NEXUS/ASTRAIA GmbH, Ismaning, Germany). An additional 131 singleton pregnancies undergoing first-trimester screening were included prospectively within a doctoral study, for which extended clinical data were available.

Pregnancy outcomes were retrieved from medical records for both components, using the same predefined clinical outcome fields whenever available. Eligible pregnancies were singleton pregnancies with first-trimester screening data sufficient for phenotype derivation. Multiple pregnancies were excluded from the analysis dataset.

The objective was to identify clinically meaningful pregnancy phenotypes using unsupervised machine learning based exclusively on maternal demographic, clinical, biophysical, and biochemical characteristics available early in pregnancy. Pregnancy, delivery, and neonatal outcomes were excluded from phenotype derivation and were used only for post hoc association analyses.

**Figure 1:**
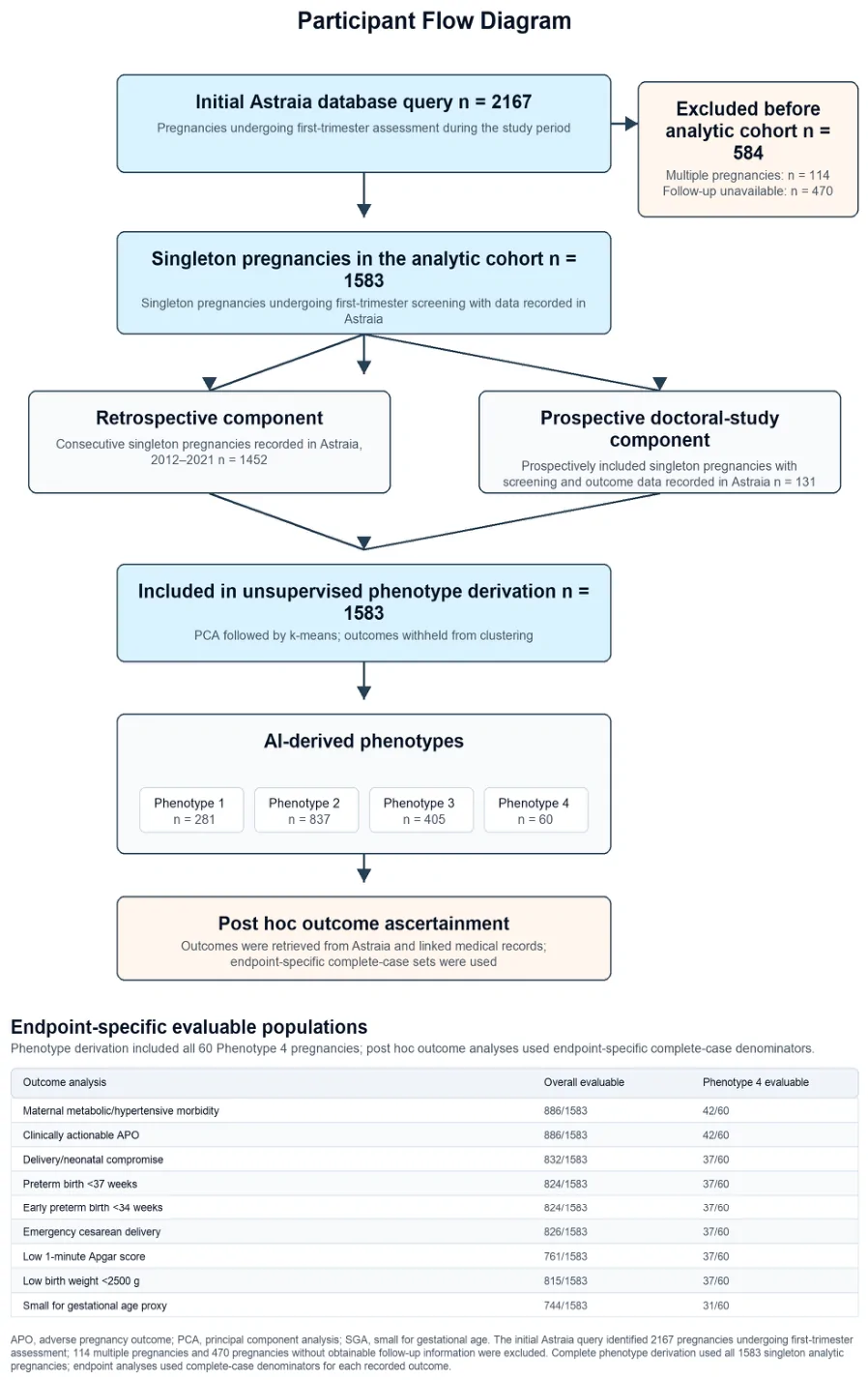
Participant flow diagram.

The cohort flow and outcome-specific analysis denominators are shown in Figure 1. The initial Astraia (version 30.1.2, NEXUS/ASTRAIA GmbH, Ismaning, Germany). database query identified 2167 pregnancies undergoing first-trimester assessment during the study period. Of these, 114 multiple pregnancies were excluded and 470 pregnancies were excluded because follow-up information could not be obtained, leaving 1583 singleton pregnancies in the analytic cohort.

Variables included in phenotype derivation were derived from routinely available first-trimester variables selected for their clinical relevance, including maternal age, gravidity, parity, body mass index (BMI), conception method, smoking status, pre-existing diabetes mellitus, chronic hypertension, previous preeclampsia, crown-rump length (CRL), nuchal translucency (NT), gestational age at examination, mean arterial pressure (MAP), mean uterine artery pulsatility index (UtA-PI), and pregnancy-associated plasma protein A (PAPP-A). Variables reflecting pregnancy complications, delivery characteristics, or neonatal outcomes were excluded to prevent outcome leakage.

### 2.2. Data Preprocessing and Dimensionality Reduction

Continuous variables were screened for implausible values and winsorized at the 1st and 99th percentiles where applicable. BMI was calculated from maternal weight and height, gestational age variables were harmonized, and PAPP-A was additionally represented on the logarithmic scale because of its skewed distribution. Missing continuous values were median-imputed within the analysis pipeline and accompanied by missingness indicators where applicable. Continuous variables were standardized to zero mean and unit variance.

Categorical variables were harmonized, infrequent categories were collapsed where necessary, and categories were encoded as indicator variables before the primary principal component analysis (PCA). The final primary preprocessing pipeline generated 39 encoded features from 23 baseline first-trimester variables.

PCA was used to obtain a lower-dimensional representation of the encoded feature space before clustering. The first four principal components were retained for the primary phenotype-discovery analysis and together explained 38.1% of the total encoded-feature variance (PC1, 14.3%; PC2, 9.4%; PC3, 7.6%; PC4, 6.7%). Clustering was therefore performed in the four-dimensional principal-component score space. 

Principal-component loadings were examined to characterize the contribution of individual continuous, binary, missingness, and categorical indicator features and, specifically, to assess whether rare binary variables such as chronic hypertension disproportionately influenced the retained PCA representation.

### 2.3. Primary Phenotype Discovery and Cluster-Number Selection

Phenotypes were derived using k-means clustering applied to the first four PCA scores. Candidate solutions with k = 2 through k = 6 was fitted using the same preprocessing and PCA framework. Candidate solutions were compared using within-cluster sum of squares (WCSS/inertia), mean silhouette coefficient, Calinski–Harabasz index, Davies–Bouldin index, cluster-size distribution, and subsampling stability.

K-means clustering used Euclidean distance in the four-dimensional PCA score space, k-means++ initialization, 30 random starts, a maximum of 300 iterations, and random seed 20260708.

Because no single quantitative index uniquely identified an optimal value of k, the final number of clusters was selected after joint consideration of internal validity indices, fixed-k reproducibility, cluster-size plausibility, and outcome-independent clinical interpretability of baseline first-trimester profiles. Pregnancy outcomes were not inspected during cluster-number selection. The gap statistic was not part of the original model-selection framework and was therefore not introduced retrospectively as a decisive criterion.

To examine the choice between the four- and five-cluster solutions, the k = 4 and k = 5 partitions were additionally compared using baseline clustering variables only. This analysis was used to determine whether the additional k = 5 cluster represented a clinically distinct baseline pattern or further subdivision of an existing phenotype.

### 2.4. Fixed-k Stability Analysis

Internal cluster stability was evaluated separately for each candidate value of k using 200 random subsamples containing 80% of the full cohort. For each subsample, preprocessing, PCA, and k-means clustering were refitted while the candidate number of clusters was held fixed. Agreement between the refitted subsample solution and the corresponding full-cohort fixed-k solution restricted to the same observations was quantified using the adjusted Rand index (ARI).

Median ARI, interquartile range, 5th–95th percentile range, and minimum–maximum values were reported for each candidate solution. The fixed-k procedure was used in the final analysis to ensure that stability estimates were directly comparable across candidate values of k.

### 2.5. Sensitivity Analyses for Preprocessing and Mixed-Data Structure

Because standardization of binary indicators and Euclidean k-means may influence clustering of mixed clinical data, focused sensitivity analyses were performed to evaluate the dependence of the primary partition on preprocessing choices and distance structure.

First, PCA loadings were examined across the retained components, with particular attention to rare binary variables, chronic hypertension, previous-preeclampsia history, and other categorical indicators. The PCA + k-means pipeline was then repeated after excluding hypertensive-history variables. Additional analyses retained binary indicators on their original 0/1 scale, down-weighted binary indicator variables, and used k − 1 reference coding for categorical variables while eliminating duplicated representation of obstetric-history information.

Agreement between each alternative preprocessing solution and the primary four-cluster solution was evaluated using global ARI and normalized mutual information (NMI). Because Phenotype 4 was the principal risk-enriched subgroup identified in the primary analysis, secondary Phenotype 4-specific recovery measures included overlap with the original phenotype, sensitivity, specificity, PPV, and Jaccard similarity.

To evaluate the effect of using a mixed-data distance metric, clustering was additionally performed using Gower dissimilarity with partitioning around medoids (PAM). Both a forced four-cluster solution and a silhouette-selected solution were evaluated. These analyses were treated as sensitivity analyses of cluster structure rather than as clinical or external validation. The final results demonstrate that preprocessing and distance specification influence the exact partition boundaries.

Because chronic hypertension was concentrated in Phenotype 4, an additional within-phenotype analysis compared patients with and without pre-existing chronic hypertension to determine whether the observed phenotype represented chronic hypertension alone or a broader cardiometabolic–vascular profile.

### 2.6. Cross-Algorithm Agreement

Reproducibility of the complete four-cluster structure across alternative unsupervised algorithms was assessed using a diagonal Gaussian mixture model and two-stage Ward hierarchical clustering. Agreement with the primary PCA + k-means partition was quantified at the complete partition level using ARI and adjusted mutual information (AMI).

Because numerical cluster labels are arbitrary, alternative-algorithm labels were aligned with primary phenotype labels using maximum-overlap assignment for presentation of contingency tables and phenotype-specific summaries only. ARI and AMI were calculated from the original partitions before relabeling and were therefore label-invariant.

Phenotype 4-specific overlap, sensitivity, specificity, PPV, and Jaccard similarity were reported as secondary subgroup-recovery measures. These analyses assessed cross-algorithm agreement and were not interpreted as validation of the primary PCA + k-means solution as a clinical reference standard.

### 2.7. Outcome Ascertainment and Definitions

Pregnancy outcomes were obtained from Astraia and linked medical records. Outcome availability was assessed separately for each endpoint according to the source variables required to derive that endpoint. Consequently, endpoint-specific complete-case denominators were used for post hoc outcome analyses, and these denominators were reported.

Chronic hypertension was defined as hypertension present before pregnancy or diagnosed before 20 weeks of gestation. Gestational hypertension was defined as new-onset systolic blood pressure ≥ 140 mmHg and/or diastolic blood pressure ≥ 90 mmHg after 20 weeks of gestation in a previously normotensive pregnancy, documented on two measurements at least 4 h apart. Preeclampsia was distinguished from gestational hypertension by the presence of proteinuria and/or other maternal or uteroplacental dysfunction. In women with pre-existing chronic hypertension who subsequently developed preeclampsia, the diagnosis was classified as preeclampsia superimposed on chronic hypertension. Preeclampsia and superimposed preeclampsia were present in the source data but occurred in only a small number of cases and were therefore not analyzed as separate phenotype-specific outcomes.

Gestational diabetes mellitus was defined according to documented clinical diagnosis based on a 75-g oral glucose tolerance test. Preterm birth was defined as delivery before 37 completed weeks of gestation and early preterm birth as delivery before 34 completed weeks. Emergency cesarean delivery was defined as cesarean delivery performed after the onset of labor or because maternal or fetal status required unplanned expedited delivery. Low birth weight was defined as birth weight < 2500 g, and low neonatal adaptation as a 1-min Apgar score < 7.

### 2.8. Composite Outcomes and Adverse-Event Burden

The maternal metabolic or hypertensive complications composite included gestational hypertension and gestational diabetes mellitus. The delivery or neonatal compromise composite included early preterm birth before 34 weeks, emergency cesarean delivery, and a 1-min Apgar score < 7.

The clinically actionable adverse pregnancy outcome (APO) composite was defined as the occurrence of at least one of the following: gestational hypertension, gestational diabetes mellitus, early preterm birth before 34 weeks, emergency cesarean delivery, or 1-min Apgar score < 7.

The expanded clinically actionable adverse obstetric outcome was defined as the clinically actionable adverse obstetric outcome additionally incorporating preterm birth before 37 completed weeks or low birth weight < 2500 g.

Cumulative morbidity was additionally summarized using the prespecified weighted adverse-event burden score and the unweighted number of adverse events per pregnancy. The proportions of pregnancies with burden scores ≥ 2 and ≥3 were evaluated across phenotypes.

### 2.9. Phenotype–Outcome Association Analyses

Outcome frequencies were first compared across all four phenotypes using endpoint-specific available denominators. Because Phenotype 4 demonstrated the greatest adverse-outcome enrichment, secondary analyses compared Phenotype 4 with Phenotypes 1–3 combined.

Relative risks (RRs) with 95% confidence intervals were estimated for principal maternal, obstetric, delivery, and neonatal outcomes. Multiple testing across related comparisons was addressed using the Benjamini–Hochberg false discovery rate procedure, with FDR-adjusted q-values reported alongside nominal *p*-values.

E-values were calculated for the principal Phenotype 4 associations to quantify the minimum strength of association that an unmeasured confounder would need to have with both phenotype membership and the outcome, conditional on measured factors, to explain the observed association or its confidence-limit estimate.

### 2.10. Missing Outcome Data and Inverse Probability Weighting

Phenotype derivation used all 1583 pregnancies and did not incorporate maternal, delivery, or neonatal outcome variables. Post hoc outcome analyses used endpoint-specific complete-case populations because outcome ascertainment depended on the availability of the source variables required for each endpoint.

Missingness in clustering variables was summarized before imputation. Outcome ascertainment was assessed by comparing pregnancies with and without documentation of the principal maternal metabolic/hypertensive endpoint. Comparisons included maternal age, BMI, gravidity, parity, MAP, UtA-PI, PAPP-A, NT, conception method, smoking, pre-existing diabetes, chronic hypertension, previous preeclampsia, and phenotype distribution. Continuous variables were compared using permutation-based median-difference tests, categorical variables using chi-square tests, and standardized differences were calculated to quantify the magnitude of imbalance.

Inverse probability weighting (IPW) was performed as a sensitivity analysis for measured predictors of principal maternal-outcome documentation. The probability of documentation was modeled using L2-penalized logistic regression including maternal age, BMI, gravidity and its missingness indicator, parity, conception method, smoking, pre-existing diabetes, chronic hypertension, and phenotype indicators. Previous preeclampsia was not included in the final IPW model.

Stabilized inverse probability weights were calculated. Predicted documentation probabilities were clipped to 0.05–0.99, with no additional truncation of the resulting weights. Weight diagnostics included the minimum, 1st, 5th, median, mean, 95th, and 99th percentiles, maximum weight, predicted-probability range, and effective sample size. Covariate balance before and after weighting was assessed using standardized differences.

Weighted risks and relative risks were estimated within the corresponding endpoint-specific documented populations. Robust sandwich-type standard errors based on the influence function for weighted risk ratios were calculated, and 2000 nonparametric bootstrap repetitions were used to provide sensitivity confidence intervals. Complete-case and IPW estimates were reported side by side.

IPW analyses were interpreted under a missing-at-random assumption conditional on the measured covariates and not as correction for possible missing-not-at-random outcome ascertainment.

### 2.11. Exploratory Risk-Enrichment Characteristics and Decision-Curve Analysis

For selected maternal and perinatal outcomes, Phenotype 4 membership was evaluated descriptively as a risk-enrichment marker. Sensitivity, specificity, PPV, negative predictive value (NPV), positive and negative likelihood ratios, risk within Phenotype 4, risk within the remaining phenotypes, and absolute risk difference were calculated using endpoint-specific denominators.

These measures were intended to characterize the distribution of adverse events within the identified phenotype and were not interpreted as evidence of a clinically validated screening or triage tool.

Decision-curve analysis was retained as an exploratory analysis of net benefit over prespecified probability thresholds. Because the study was single-center, outcome ascertainment was incomplete, sensitivity of Phenotype 4 was low, and no external validation was available, decision-curve results were not considered evidence supporting phenotype-guided surveillance or intervention.

### 2.12. Additional Subgroup and Sensitivity Analyses

Phenotype–outcome associations were additionally examined in prespecified clinically relevant subsets, including nulliparous pregnancies, spontaneously conceived pregnancies, and pregnancies with available PAPP-A measurements.

Sensitivity analyses also included the alternative preprocessing and mixed-data clustering approaches described above, fixed-k subsampling stability, comparison of the four- and five-cluster solutions, the chronic-hypertension stratification within Phenotype 4, and IPW analyses for incomplete outcome ascertainment.

### 2.13. Incremental Value of Phenotype Membership

The incremental contribution of AI-derived phenotype membership beyond routinely available clinical variables was explored using five-fold cross-validated predictive models. For each evaluated outcome, a clinical model was compared with the corresponding model augmented by phenotype membership. Performance was summarized using AUROC, area under the precision–recall curve (AUPRC), and Brier score, and the differences between models were reported.

These analyses were exploratory and were used to assess whether phenotype membership provided additional within-cohort discriminatory information. The incremental gain was selective and concentrated mainly in early preterm birth and low 1-min Apgar score.

### 2.14. Phenotype-Translation Analyses

Exploratory phenotype-translation analyses were performed to determine whether the primary AI-derived Phenotype 4 label could be approximated using a simplified combination of routinely available first-trimester variables. The target for both models was Phenotype 4 membership and not any maternal, obstetric, delivery, or neonatal outcome.

The logistic surrogate included maternal age, BMI, MAP, mean UtA-PI, PAPP-A, relevant missingness indicators, chronic hypertension, pre-existing diabetes, maternal family history of preeclampsia, and conception method.

Five-fold stratified cross-validation was generated once using random seed 20260708. Each held-out fold contained 12 Phenotype 4 pregnancies. Within each training fold, continuous variables were median-imputed and z-standardized, categorical variables were normalized and dummy-encoded, and the held-out matrix was aligned to the training-fold design. L2-penalized logistic regression was fitted exclusively in training folds, and performance estimates were obtained from held-out out-of-fold probabilities.

The simple points-based score assigned 1 point each for maternal age ≥ 35 years, BMI ≥ 30 kg/m^2^, MAP ≥ the training-fold 90th percentile, mean UtA-PI ≥ the training-fold 90th percentile, and PAPP-A ≤ the training-fold 10th percentile; 2 points for chronic hypertension; 1 point for pre-existing diabetes; and 2 points for previous preeclampsia.

The total possible score ranged from 0 to 10. Data-derived biomarker cutoffs were recalculated within each training fold. All five training folds independently selected the same classification threshold of ≥3 points under the specificity ≥ 0.95 selection rule; therefore, the fixed ≥ 3 threshold was applied to held-out fold scores without re-optimization.

Phenotype-replication performance was summarized using AUROC, sensitivity, specificity, PPV, and NPV. Ninety-five percent confidence intervals were calculated using 2000 patient-level nonparametric bootstrap repetitions of the final out-of-fold predictions or scores. For the points-based score, AUROC was calculated from the raw out-of-fold score.

Calibration was assessed using the Brier score, calibration intercept obtained from a logistic calibration model with slope fixed to 1, and calibration slope estimated from logistic regression of the phenotype label on the logit of predicted probability. For the points-based score, Brier score and calibration measures used fold-specific score-to-probability mappings fitted exclusively in the training folds.

### 2.15. Statistical Analysis and Reproducibility

Continuous variables are reported as median and interquartile range, and categorical variables as number and percentage. Descriptive statistics in Table 1 were calculated from observed, non-imputed values. Between-phenotype comparisons used permutation-based procedures for continuous variables and categorical tests appropriate to the distribution and cell counts. Relative risks are reported with 95% confidence intervals. Two-sided statistical testing was used, with *p* < 0.05 considered statistically significant before correction for multiple comparisons.

All analyses were performed in Python 3.12.13 (Python Software Foundation, Wilmington, DE, USA) on macOS-26.5.1-arm64 using NumPy 2.3.5, pandas 2.2.3, openpyxl 3.1.5, Pillow 12.2.0, and python-docx 1.2.0. PCA, k-means clustering, diagonal Gaussian-mixture modeling, Ward microcluster aggregation, permutation tests, bootstrap resampling, inverse probability weighting, calibration metrics, and decision-curve calculations were implemented using custom NumPy/pandas routines. SciPy, scikit-learn, statsmodels, lifelines, xgboost, and matplotlib were not used in the final Python analysis scripts.

Random seeds were fixed for all stochastic procedures; the primary analysis used seed 20260708, with deterministic offsets for candidate k values, permutation tests, cross-validation folds, bootstrap analyses, Gaussian-mixture initialization, and microcluster initialization.

## 3. Results

A comparison of maternal characteristics across the four phenotypes is presented in Table 1. Maternal age differed significantly between phenotypes (*p* < 0.001), with patients included in Phenotype 4 exhibiting the highest median age (34.4 years), followed by Phenotype 1 (33.0 years), whereas Phenotype 3 included the youngest participants (28.9 years). Body mass index also varied significantly across clusters (*p* < 0.001). Phenotype 4 was characterized by the highest BMI (28.19 kg/m^2^), while Phenotypes 2 and 3 had similar and comparatively lower median BMI values (22.04 kg/m^2^).

Significant differences were observed in obstetric history. Gravidity and parity were both higher in Phenotypes 1 and 4 (*p* < 0.001). Moreover, Phenotypes 2 and 3 consisted exclusively of nulliparous women, whereas Phenotype 1 comprised almost entirely multiparous women without a previous history of preeclampsia (99.3%). On the other hand, nearly half of the patients included in Phenotype 4 had a previous pregnancy complicated by preeclampsia (45.0%).

Although spontaneous conception predominated in all groups, assisted reproductive techniques, particularly in vitro fertilization, were more common in Phenotype 4 (13.3%) and Phenotype 2 (6.5%) than in the remaining phenotypes (*p* < 0.001).

Smoking during pregnancy was similarly distributed among phenotypes and did not reach statistical significance (*p* = 0.271). Diabetes mellitus showed a statistically significant difference across phenotypes (*p* = 0.026), but had a very low overall prevalence within the cohort. Chronic hypertension demonstrated the most pronounced between-group difference (*p* < 0.001). While it was absent in Phenotypes 1–3, more than half of the patients included in Phenotype 4 had pre-existing chronic hypertension (56.7%), highlighting this phenotype as one predominantly driven by maternal cardiovascular risk factors. On the other hand, a maternal family history of preeclampsia was infrequent and did not differ significantly among groups (*p* = 0.254).

Among first-trimester screening markers, mean arterial pressure differed markedly between phenotypes (*p* < 0.001), reaching the highest values in Phenotype 4 (101.0 mmHg). Mean uterine artery pulsatility index also differed significantly (*p* = 0.030), with the highest values observed in Phenotype 3 and the lowest in Phenotype 4.

PAPP-A concentrations varied significantly across phenotypes (*p* < 0.001). The lowest median PAPP-A value was observed in Phenotype 3 (0.75 MoM). Also, nuchal translucency measurements differed significantly (*p* < 0.001), with Phenotype 3 presenting the highest median value (1.90 MoM).

**Figure 2:**
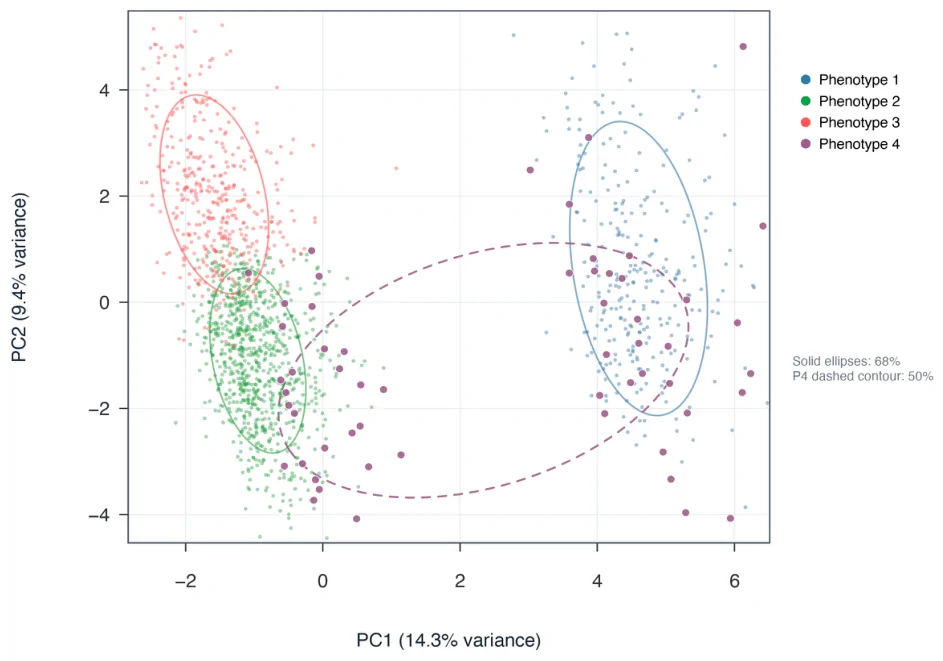
Two-dimensional PCA projection of the four-cluster solution. Each point represents one pregnancy projected onto the first two principal components. Clustering was performed in the space of the first four principal components rather than in the two-dimensional projection shown. PC1 and PC2 explain 14.3% and 9.4% of the encoded-feature variance, respectively (23.7% cumulatively), whereas the four retained principal components explain 38.1% of the total variance. Solid ellipses represent the 68% covariance ellipses for Phenotypes 1–3, whereas the dashed contour represents the 50% covariance ellipse for Phenotype 4 because of its smaller sample size.

The four-cluster solution is illustrated by a two-dimensional projection onto the first two principal components (Figure 2). PC1 and PC2 explained 14.3% and 9.4% of the total encoded-feature variance, respectively (23.7% cumulatively), whereas clustering was performed using the first four principal components, which together explained 38.1% of the variance (Supplementary Table S1). As expected for a two-dimensional projection of higher-dimensional data, partial overlap between the projected phenotypes was observed, particularly between Phenotypes 2 and 3. Accordingly, Figure 2 should be interpreted as an illustrative visualization of the clustering solution. The two-dimensional PC1–PC2 projection shown in Figure 2, which represents 23.7% of the total variance, was used as an illustrative visualization and not as an independent criterion of cluster validity.

Quantitative evaluation of the clustering solution was based on internal validity indices, fixed-k stability analyses, and principal-component loadings (Supplementary Tables S2–S5).

Among the 1583 pregnancies included in phenotype derivation, the principal maternal outcome was available for 886 pregnancies (56.0%), including 42 of 60 patients in Phenotype 4 (70.0%). Outcome availability varied across endpoints and phenotypes: maternal metabolic/hypertensive and clinically actionable adverse pregnancy outcome analyses included 886 pregnancies, delivery or neonatal compromise included 832, preterm birth outcomes included 824, emergency cesarean delivery included 826, low 1-min Apgar score included 761, low birth weight included 815, and the cohort-derived SGA proxy included 744 pregnancies (Supplementary Tables S6 and S7).

Comparison of pregnancies with and without documented principal maternal outcomes showed differences in several baseline characteristics, most notably phenotype distribution (absolute standardized difference 0.425), parity (0.369), gravidity (0.182), age (0.155), PAPP-A (0.156), chronic hypertension (0.127), conception method (0.123), and smoking (0.101) (Supplementary Table S8a).

After stabilization and propensity-score clipping, IPW weights were well behaved, with a median of 1.012, a 99th percentile of 2.412, a maximum of 3.488, and an effective sample size of 811.1 among documented pregnancies (Supplementary Table S8b). Weighting substantially improved balance across measured documentation predictors, reducing the maximum absolute standardized difference from 0.425 before weighting to 0.039 after weighting (Supplementary Table S9).

Weighted estimates were broadly consistent with complete-case results for the principal outcomes. The IPW relative risk for maternal metabolic or hypertensive complications was 3.99 (robust 95% CI 2.79–5.70), compared with a complete-case RR of 4.31 (95% CI 3.08–6.05). Similarly, the IPW RR for clinically actionable adverse pregnancy outcomes was 2.15 (robust 95% CI 1.59–2.92), compared with a complete-case RR of 2.31 (95% CI 1.74–3.07). For rarer delivery and neonatal outcomes, point estimates remained above 1 but confidence intervals widened, particularly in bootstrap analyses (Supplementary Table S10). These weighted analyses were therefore interpreted as sensitivity analyses under a missing-at-random assumption conditional on measured covariates, rather than as correction for possible missing-not-at-random outcome ascertainment.

Missingness of variables used for phenotype derivation was generally low, except for MAP-related variables (11.4%) and PAPP-A (30.3%) (Supplementary Table S11).

We first examined the contribution of individual variables to the retained principal components in order to evaluate the influence of preprocessing and variable encoding on phenotype derivation. Although rare binary indicators contributed to several principal components, chronic hypertension was not consistently among the dominant contributors across the PCA solution. Specifically, chronic hypertension ranked second and third among absolute loadings only for PC3, whereas its contribution was substantially lower in the remaining retained components (PC1 rank 22, PC2 rank 15, PC4 rank 17, PC5 rank 9, and PC6 rank 37).

On the other hand, maternal anthropometric characteristics, obstetric history, first-trimester biomarkers, and uterine artery Doppler measurements contributed across multiple principal components, indicating that the latent structure underlying Phenotype 4 was multidimensional rather than driven by a single rare binary variable (Supplementary Tables S4 and S5).

Alternative preprocessing strategies demonstrated that the overall clustering solution was sensitive to the treatment of binary and categorical variables, although a substantial proportion of the primary high-risk phenotype remained identifiable. Excluding hypertensive-history variables recovered 47 of the 60 patients assigned to the primary Phenotype 4 (sensitivity 78.3%), while retaining binary indicators as 0/1 recovered 40 of 60 patients (66.7%) and down-weighting binary variables recovered 44 of 60 patients (73.3%). A reference-coded analysis using *k* − 1 dummy variables and removal of the duplicated obstetric-history representation recovered 42 of the 60 primary Phenotype 4 patients (70.0% sensitivity); however, these patients were incorporated into a substantially larger cluster (*n* = 281), resulting in relatively low PPV (14.9%) and Jaccard similarity (0.140). Across all preprocessing strategies, global agreement with the primary clustering solution remained modest (ARI 0.14–0.20), indicating that preprocessing influenced the exact boundaries of Phenotype 4 while preserving a substantial proportion of the underlying cardiometabolic signal (Supplementary Table S12).

To evaluate whether the Euclidean distance assumption influenced phenotype derivation, clustering was repeated using Gower dissimilarity with partitioning around medoids (PAM). In the forced four-cluster solution, the cluster showing the greatest similarity to the primary Phenotype 4 contained 19 patients and recovered 11 of the 60 original Phenotype 4 patients (sensitivity 18.3%), while maintaining very high specificity (99.5%) and PPV (57.9%). The silhouette-selected two-cluster solution recovered a larger proportion of Phenotype 4 (26/60; sensitivity 43.3%) but showed substantially lower specificity and PPV. These findings indicate that mixed-data clustering identified a narrower subgroup with a similar cardiometabolic risk profile rather than reproducing the complete primary Phenotype 4, supporting the interpretation that the high-risk signal is preserved despite changes in distance metric and clustering methodology (Supplementary Table S13).

To further examine whether Phenotype 4 merely reflected pre-existing chronic hypertension, outcomes were compared between patients with and without chronic hypertension within this phenotype. Adverse pregnancy outcomes remained frequent in both subgroups, although absolute event rates were generally higher among patients with chronic hypertension, particularly for gestational hypertension (34.6% vs. 18.8%) and clinically actionable adverse pregnancy outcomes (65.4% vs. 43.8%). These findings suggest that chronic hypertension contributes importantly to the phenotype but does not fully explain its adverse outcome profile, supporting the interpretation of Phenotype 4 as a broader cardiometabolic–vascular risk-enrichment phenotype rather than a simple surrogate for chronic hypertension alone (Supplementary Table S14).

Candidate cluster solutions showed partial disagreement across internal validation criteria. The two-cluster solution achieved the highest silhouette coefficient and Calinski–Harabasz index and the lowest Davies–Bouldin index, indicating the strongest purely geometric separation but a clinically coarse partition. Although the five-cluster solution demonstrated higher fixed-k stability and more favorable Calinski–Harabasz and Davies–Bouldin indices than the four-cluster solution, direct comparison showed that it did not identify a clearly distinct additional baseline phenotype. Phenotype 1 was preserved completely (281/281 patients), and Phenotype 4 was almost entirely retained (59/60 patients), whereas the principal difference was subdivision of the nulliparous population already represented by Phenotypes 2 and 3. Consequently, the four-cluster solution was retained as the most intermediate representation balancing quantitative performance, cluster-size plausibility, and clinically coherent baseline phenotypes identified without using pregnancy outcomes (Supplementary Tables S1–S3 and S15).

Pregnancy outcomes across the four phenotypes are presented in Table 2. Adverse obstetric outcomes were consistently more frequent in Phenotype 4. Clinically actionable adverse pregnancy outcomes occurred in 57.1% of pregnancies assigned to Phenotype 4, compared with 23.6–31.9% across the other phenotypes (*p* < 0.001). The expanded clinically actionable adverse pregnancy outcome was also most frequent in Phenotype 4 (59.5% vs. 27.4–33.0%; *p* < 0.001). Within Phenotype 4, 28.6% of pregnancies met criteria for the maternal metabolic or hypertensive complications composite only, 4.8% met criteria for the delivery or neonatal compromise composite only, and 23.8% met criteria for both composites.

The incidence of gestational hypertension differed significantly among phenotypes (*p* < 0.001), with the highest frequency observed in Phenotype 4 (28.6%), compared with 7.9% in Phenotype 3, 5.5% in Phenotype 2, and 1.1% in Phenotype 1. Gestational diabetes mellitus also differed significantly among phenotypes (*p* < 0.001), occurring most frequently in Phenotype 4 (33.3%) and Phenotype 1 (17.0%), compared with 7.1% and 4.6% in Phenotypes 2 and 3, respectively. Accordingly, the maternal metabolic or hypertensive complications composite was most frequent in Phenotype 4 (52.4% vs. 10.4–18.1%; *p* < 0.001).

Although preterm birth before 37 weeks was numerically most frequent in Phenotype 4 (16.2%), the difference across phenotypes was not statistically significant (*p* = 0.154). In contrast, early preterm birth before 34 weeks differed significantly among phenotypes (*p* = 0.034), with the highest rate observed in Phenotype 4 (10.8%). Emergency cesarean delivery was also most frequent in Phenotype 4 (27.0%), followed by Phenotype 1 (16.7%), whereas Phenotypes 2 and 3 had similar rates of approximately 11% (*p* = 0.020).

Neonatal adaptation, assessed using the 1-min Apgar score, differed significantly among phenotypes (*p* = 0.009). A 1-min Apgar score < 7 occurred most frequently in Phenotype 4 (8.1%) and Phenotype 3 (5.6%), whereas no cases were observed in Phenotype 1. The delivery or neonatal compromise composite was likewise more frequent in Phenotype 4 (32.4% vs. 14.1–17.9%; *p* = 0.029).

Low birth weight < 2500 g and small for gestational age < 10th percentile did not differ significantly among phenotypes (*p* = 0.388 and *p* = 0.631, respectively), despite the numerically higher rate of low birth weight in Phenotype 4. Similarly, the composite adverse fetal outcome did not differ significantly across phenotypes (*p* = 0.136).

The cumulative burden of adverse pregnancy outcomes also differed substantially across phenotypes. Phenotype 4 had the highest mean weighted adverse burden score (1.29 vs. 0.35–0.47; *p* < 0.001) and the highest mean adverse event count (1.12 vs. 0.31–0.38; *p* < 0.001). The proportion of pregnancies with a burden score ≥ 2 was highest in Phenotype 4 (28.6% vs. 4.3–12.4%; *p* < 0.001), while a burden score ≥ 3 occurred in 9.5% of Phenotype 4 pregnancies compared with 1.8–4.6% in the remaining phenotypes (*p* = 0.014) (Table 2).

We further performed relative risk analyses by comparing this subgroup with the remaining phenotypes combined to further quantify the excess risk associated with Phenotype 4 (Table 3). Gestational hypertension was over five times more frequent among patients with Phenotype 4 than among patients in the remaining phenotypes (RR = 5.07, 95% CI 2.95–8.70, *p* < 0.001), while the risk of gestational diabetes mellitus was increased approximately 4.5-fold (RR = 4.49, 95% CI 2.77–7.26, *p* < 0.001). Likewise, the composite outcome of maternal metabolic or hypertensive complications was associated with a more than fourfold increased risk (RR = 4.31, 95% CI 3.08–6.05, *p* < 0.001).

Phenotype 4 was also associated with a significantly greater likelihood of clinically relevant adverse pregnancy outcomes. Patients assigned to this phenotype had more than twice the risk of experiencing a clinically actionable adverse pregnancies outcomes (RR = 2.31, 95% CI 1.74–3.07, *p* < 0.001) and an expanded clinically actionable adverse pregnancy outcome (RR = 2.10, 95% CI 1.60–2.75, *p* < 0.001) compared with patients in the other phenotypes.

Regarding perinatal outcomes, Phenotype 4 was associated with a significantly increased risk of delivery or neonatal compromise (RR = 2.14, 95% CI 1.32–3.46, *p* = 0.029), early preterm birth before 34 weeks (RR = 3.97, 95% CI 1.53–10.32, *p* = 0.035), emergency cesarean section (RR = 2.36, 95% CI 1.36–4.09, *p* = 0.022), and a 1-min Apgar score below 7 (RR = 3.26, 95% CI 1.10–9.65, *p* = 0.012).

All statistically significant associations remained significant after false discovery rate correction, with *q* values ranging from <0.001 to 0.039. Furthermore, E-values ranged from 3.62 to 9.60, suggesting that relatively strong unmeasured confounding would be required to fully explain the observed associations. The largest E-values were observed for gestational hypertension (9.60), gestational diabetes mellitus (8.44), and the composite maternal metabolic or hypertensive complications outcome (8.09) (Table 3).

**Figure 3:**
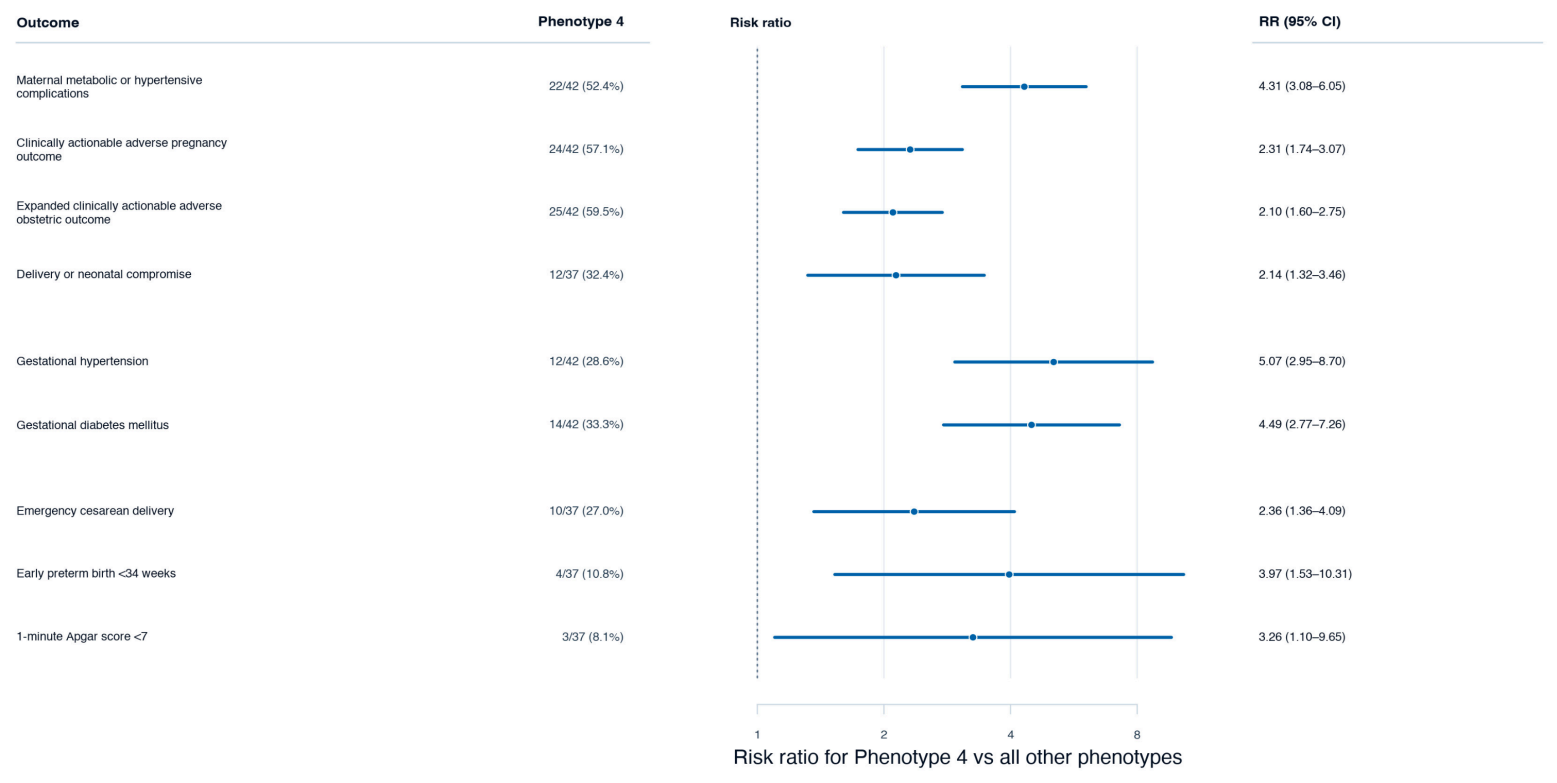
Phenotype 4 associations with adverse obstetric outcomes.

The forest plot shows that Phenotype 4 was consistently enriched for clinically relevant adverse outcomes compared with the remaining phenotypes (Figure 3). The strongest associations were observed for maternal metabolic or hypertensive complications, gestational hypertension, gestational diabetes, clinically actionable adverse pregnancy outcomes, emergency cesarean delivery, early preterm birth, and low Apgar score. The pattern of association supports a predominantly maternal cardiometabolic–vascular risk phenotype, with secondary enrichment for delivery and neonatal compromise, rather than a pure fetal-growth-restriction phenotype. Also, this phenotype with the highest adverse-event burden, whose defining baseline characteristics were predominantly maternal cardiometabolic and vascular did not comprise a pattern dominated by abnormal placental biomarkers.

Table 4 was evaluated for a range of clinically relevant maternal and perinatal outcomes (Table 4). For maternal metabolic or hypertensive complications, Phenotype 4 achieved the highest predictive performance, with a specificity of 97.4%, a PPV of 52.4%, and a positive likelihood ratio (LR+) of 6.76. Patients classified as Phenotype 4 had an absolute risk of 52.4% compared with 12.1% among patients in the remaining phenotypes, corresponding to an absolute risk difference of 40.3%.

Similarly, Phenotype 4 demonstrated good predictive performance for clinically actionable adverse pregnancy outcomes. The PPV was 57.1% for clinically actionable APOs and 59.5% for the expanded composite APO, with specificities exceeding 97% for both outcomes. Absolute risk differences reached 32.5% and 31.3%, respectively.

Performance was more modest for neonatal outcomes. For delivery or neonatal compromise, the PPV was 32.4%, with a specificity of 96.4% and an LR+ of 2.50. Early preterm birth before 34 weeks demonstrated a relatively low PPV (10.8%) despite a high specificity (95.9%), reflecting the low baseline prevalence of this outcome within the cohort. Similar findings were observed for emergency cesarean section (PPV 27.0%) and a 1-min Apgar score below 7 (PPV 8.1%), although both outcomes retained high NPVs (>88%) and specificities exceeding 95%.

Across all evaluated outcomes, sensitivity remained relatively low (9.0–17.7%), indicating that Phenotype 4 identified only a subset of patients who subsequently experienced adverse outcomes. On the other hand, specificity consistently exceeded 95%, and NPVs ranged from 71.8% to 97.2%, demonstrating that patients not classified as Phenotype 4 generally had a lower probability of adverse events.

**Figure 4:**
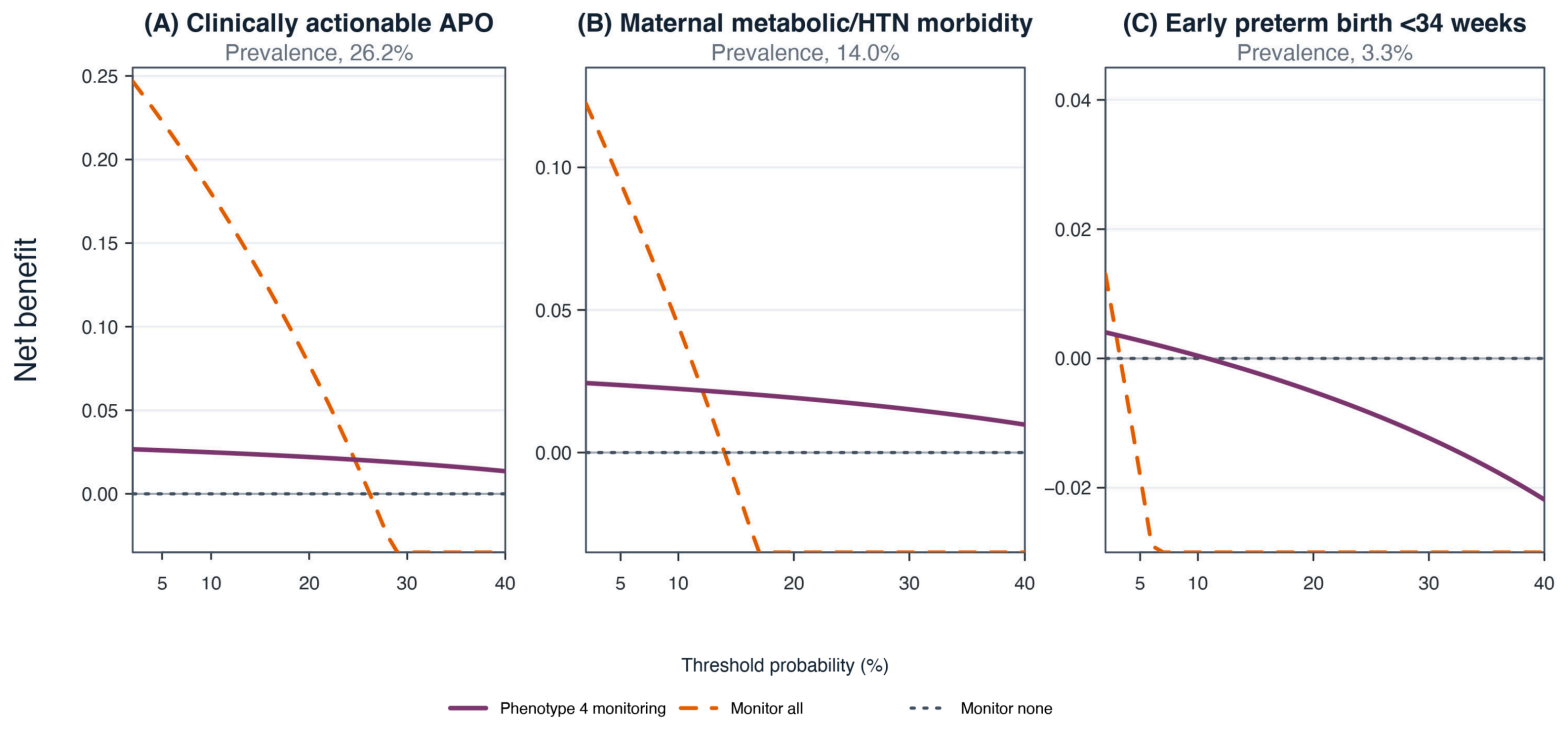
Decision curve analysis for Phenotype 4-triggered monitoring. (**A**) Clinically actionable adverse pregnancy outcome. (**B**) Maternal metabolic or hypertensive morbidity. (**C**) Early preterm birth before 34 completed weeks.

Decision curve analysis showed the potential clinical net benefit of a phenotype-triggered monitoring strategy (Figure 4). Across clinically relevant threshold probabilities, identifying Phenotype 4 as a subgroup for intensified surveillance provided higher net benefit than default strategies in selected outcome settings, particularly for clinically actionable APO and maternal metabolic-hypertensive complications. This supports the clinical utility of the phenotype as a risk-enrichment strategy rather than as a universal prediction model.

Sensitivity analyses restricted to nulliparous pregnancies, spontaneously conceived pregnancies, and pregnancies with available PAPP-A measurements yielded similar estimates, supporting the robustness of the primary findings (Supplementary Table S16). Outcome estimates obtained after inverse probability weighting were similar to the primary analysis, suggesting that incomplete outcome ascertainment had minimal influence on the observed associations (Supplementary Table S17).

Cross-algorithm agreement was assessed at the level of the complete four-cluster partition, with the primary PCA + k-means solution used only as the reference partition for algorithmic comparison rather than as a clinical gold standard (Table 5). Agreement with the primary solution was higher for the diagonal Gaussian mixture model (ARI 0.763; AMI 0.770) than for the two-stage Ward hierarchical clustering solution (ARI 0.660; AMI 0.695).

In secondary Phenotype 4-specific recovery analyses, the Gaussian mixture Phenotype 4-matched cluster included 71 patients and overlapped with 60/60 primary Phenotype 4 patients (sensitivity 1.000, specificity 0.993, PPV 0.845, Jaccard similarity 0.845), whereas the Ward Phenotype 4-matched cluster included 59 patients and overlapped with 56/60 primary Phenotype 4 patients (sensitivity 0.933, specificity 0.998, PPV 0.949, Jaccard similarity 0.889).

Separately, phenotype-translation models designed to approximate Phenotype 4 membership achieved AUROC values of 0.887 for the cross-validated logistic surrogate model and 0.884 for the simple points-based score (≥3). These models should be interpreted as tools for approximating phenotype membership.

Supplementary Tables S18–S21 report the leakage-safe internal performance and calibration of the logistic surrogate and simple points-based score, the complete scoring system, the logistic model specification and descriptive equation, and the cross-validation structure. Both models were developed exclusively to approximate membership in the AI-derived Phenotype 4. Because their predictors overlap with variables contributing to phenotype derivation, these analyses represent phenotype replication rather than independent clinical outcome prediction. All reported performance estimates were derived from held-out out-of-fold predictions or scores, with data-dependent preprocessing and threshold derivation performed within training folds.

Addition of AI-derived phenotype membership produced modest changes in predictive discrimination compared with conventional clinical models. Improvements were observed primarily for rare neonatal outcomes, including early preterm birth (ΔAUROC = +0.085) and low 1-min Apgar score (ΔAUROC = +0.093), whereas discrimination for more common maternal outcomes remained largely unchanged (Supplementary Table S22). Bootstrap validation demonstrated consistent estimates across repeated resampling (Supplementary Table S23).

**Figure 5:**
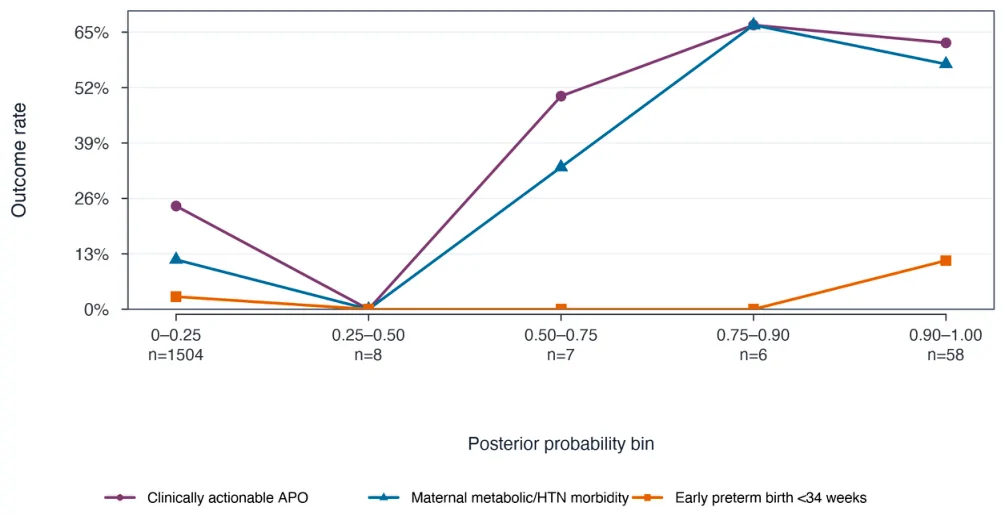
Outcome gradient by GMM posterior probability.

Figure 5 evaluates phenotype assignment uncertainty using Gaussian-mixture posterior probabilities. Patients with the highest posterior probability of belonging to the phenotype-4-like cluster had the highest rates of clinically actionable adverse pregnancy outcomes, maternal metabolic-hypertensive complications, and early preterm birth. This probability-response pattern supports the biological and clinical coherence of the phenotype.

## 4. Discussion

In an unselected cohort of 1583 first-trimester singleton pregnancies, unsupervised clustering of routinely available maternal, biophysical, and biochemical variables reproducibly identified four latent phenotypes. One of these, Phenotype 4, representing fewer than 5% of the cohort, concentrated a disproportionate share of subsequent adverse events: the risk of gestational hypertension was increased approximately five-fold (RR 5.07, 95% CI 2.95–8.70), the risk of gestational diabetes 4.5-fold, and the risks of early preterm birth, emergency caesarean delivery, and 1-min Apgar score below 7 were each significantly higher than in the pooled remaining phenotypes.

More than half of patients included in Phenotype 4 experienced a clinically actionable adverse pregnancy outcome, all associations remained significant after false-discovery-rate correction, and E-values between 3.6 and 9.6 indicated that only substantial unmeasured confounding could explain them away.

The phenotype was recovered with sensitivities of 93–100% and Jaccard indices of 0.85–0.89 by two independent clustering algorithms (diagonal Gaussian mixture model and two-stage Ward hierarchical clustering), was reproduced across sensitivity analyses in nulliparous, spontaneously conceived, and PAPP-A–available subsets, and could be approximated by a cross-validated logistic surrogate model with an AUROC of 0.91 and by an interpretable points-based score.

The maternal profile of Phenotype 4 characterized by older patients, higher BMI, chronic hypertension in more than half of the group, elevated mean arterial pressure, more frequent previous preeclampsia, and a higher prevalence of assisted conception is consistent with a cardiometabolic–vascular substrate rather than a placental-insufficiency or fetal-growth-restriction phenotype. This interpretation is supported by the pattern of outcomes in which enrichment was strongest for hypertensive and metabolic complications and their downstream delivery/neonatal consequences, whereas mean uterine artery pulsatility index was not elevated and small-for-gestational-age rates did not differ significantly across phenotypes. Thus, the phenotype with the greatest adverse-outcome burden was characterized predominantly by maternal cardiometabolic and vascular vulnerability rather than by an isolated placental biomarker pattern.

The clinical and biological logic aligns with a substantial literature demonstrating that pre-pregnancy cardiometabolic risk factors cluster and act synergistically to drive adverse pregnancy outcomes, that this clustering intensifies with advancing maternal age, and that maternal obesity, chronic hypertension, and glucose intolerance share overlapping vascular and inflammatory mechanisms that lead to both hypertensive disorders and gestational diabetes [1,16,27]. In this framework, Phenotype 4 is best understood as the first-trimester crystallisation of an integrated maternal cardiometabolic phenotype, whose clinical signature emerges months before the classical placental syndrome becomes overt.

This interpretation is also supported by multimorbidity analyses performed in unselected obstetric populations. Garcia and colleagues identified latent maternal classes characterized by obesity, chronic hypertension, advanced maternal age and assisted reproduction, demonstrating that these characteristics naturally cluster rather than acting independently [28]. Their cardiometabolic class resembles our Phenotype 4, whereas their reproductive-history class parallels the predominantly nulliparous profile observed in our lower-risk phenotypes, suggesting that our findings reflect broader maternal health patterns rather than disease-specific clustering alone.

Our findings extend, rather than replicate, the existing unsupervised-phenotyping literature in pregnancy. Prior clustering studies have almost invariably been conducted downstream of a diagnosis in cohorts of women already carrying preeclampsia, gestational diabetes, or preterm birth, and have used the joint structure of clinical, biomarker, or histopathological data to refine that single condition into subtypes.

Houri and colleagues, for example, applied UMAP and k-means to 482 women with preeclampsia and identified three phenotypes with maternal complication rates ranging from 4% to 23% [21]. In their study, the authors found three PE phenotypes, and the most prevalent was Cluster A (46.2% of patients) which comprised patients with the highest rates of preterm delivery, angiogenic imbalance, fetal growth restriction and maternal and neonatal complications [21]. Zhu and colleagues defined four data-driven gestational diabetes clusters with differential risks of perinatal complications and postpartum diabetes. The highest-risk phenotype, characterized by early GDM diagnosis, pre-existing comorbidities, and marked hyperglycemia during glucose challenge testing, showed significantly increased risks of severe maternal morbidity, neonatal intensive care unit admission, and a more than fourfold higher incidence of postpartum diabetes compared with the reference cluster [24].

Our study departs from these designs on two axes. First, phenotyping was performed at 11–13 weeks, in an unselected first-trimester population, using only variables available before any adverse outcome had occurred. Second, the phenotypes stratify risk across the full spectrum of maternal and perinatal outcomes rather than refining a single diagnosis.

The ability to recover clinically meaningful phenotypes before overt disease develops is supported by recent first-trimester machine-learning studies. Horgan and colleagues demonstrated that unsupervised clustering among patients with apparently normal first-trimester blood pressure identified a small subgroup with an eight-fold higher risk of subsequent preeclampsia despite remaining below conventional diagnostic thresholds [29]. This observation reinforces the concept that latent vascular risk is detectable early in pregnancy and supports our finding that first-trimester variables can identify clinically relevant phenotypes before pregnancy complications become clinically manifest.

Placental pathology studies provide complementary evidence that adverse pregnancy phenotypes are biologically heterogeneous. Using K-modes clustering of maternal vascular malperfusion lesions, Zur and colleagues identified distinct placental pathological phenotypes, including separate decidual vasculopathy and accelerated villous maturation clusters with different associations with preeclampsia, fetal growth restriction and preterm birth [30]. These findings support our observation that maternal cardiometabolic burden and markers of placental dysfunction do not necessarily coexist within the same phenotype.

The magnitudes we observed for Phenotype 4 (approximately five-fold risk for gestational hypertension and 4.5-fold for gestational diabetes) are consistent with the highest-risk clusters reported in downstream-phenotyping studies, and are of the same order as effect sizes reported for chronic hypertension alone in large meta-analyses (adjusted OR 5.43 for preeclampsia, 2.23 for preterm birth) [17]. This suggests that the phenotype captures a maternal risk substrate whose magnitude is comparable to that of chronic hypertension, but that additionally integrates BMI, previous preeclampsia, mean arterial pressure, and conception mode into a single latent construct.

The diagnostic characteristics of Phenotype 4 should be interpreted as descriptive characteristics of an exploratory risk-enriched subgroup rather than evidence supporting a clinical triage strategy. Sensitivity was modest (9–17.7%), reflecting the small size of the phenotype, whereas specificity consistently exceeded 95% and positive predictive values for the principal composite outcomes were above 50%. These findings indicate enrichment of adverse outcomes within the identified subgroup but do not establish clinical utility or justify implementation of phenotype-guided surveillance.

Decision-curve analyses were exploratory and should not be interpreted as demonstrating clinical benefit. Rather, they suggest that the identified phenotype may warrant further evaluation in prospective validation studies. Importantly, Phenotype 4 should not be viewed as an alternative to established outcome-specific prediction models, such as the Fetal Medicine Foundation competing-risks model. Instead, if externally validated, multidimensional phenotyping could be explored as a complementary approach for characterizing maternal risk profiles beyond individual pregnancy outcomes.

Our findings are also consistent with studies demonstrating that latent pregnancy phenotypes are better characterised by the cumulative burden of adverse outcomes than by isolated complications. Latent class analyses in women with preeclampsia have shown that high-risk phenotypes accumulate persistent postpartum hypertension together with more severe antenatal disease, while large population-based analyses have identified multimorbidity clusters combining hypertension, diabetes and other maternal disorders that are associated with substantially higher intervention rates, including caesarean delivery [31,32].

Several properties of the analysis support these findings. Repeated 80% subsampling yielded a median adjusted Rand index of 0.90, in the same range as the 0.88 reported for organ-system-based preeclampsia clustering in a considerably larger cohort. Cross-algorithm agreement with Gaussian mixture modelling and Ward hierarchical clustering recovered Phenotype 4 with high sensitivity, specificity, and Jaccard similarity, indicating that the subgroup is not an artifact of the primary PCA-plus-k-means pipeline. Sensitivity analyses restricted to nulliparous, spontaneously conceived, and PAPP-A–available subsets, together with inverse probability weighting for incomplete outcome ascertainment, produced concordant estimates. Posterior probabilities from the Gaussian mixture model showed a monotonic dose–response relationship with outcome frequency, an internal-consistency signal characteristic of internally coherent clustering solution phenotypes rather than threshold artifacts. Finally, E-values in the 3.6–9.6 range place the observed associations well beyond the range that could be neutralised by plausible unmeasured confounders.

Exploratory phenotype-translation analyses suggested that the AI-derived Phenotype 4 label could be approximated using routinely available first-trimester variables. However, these analyses were designed to reproduce phenotype membership rather than predict obstetric outcomes and are therefore partly circular by design. Consequently, the reported discrimination metrics should be interpreted as measures of phenotype replication rather than clinical prediction performance. External validation is required before any phenotype-translation tool can be considered for clinical use.

Strengths include a reasonably sized unselected first-trimester cohort, the exclusive use of variables already collected in routine practice, blinding of outcomes during phenotype derivation, cross-algorithm and cross-subsample agreement, and explicit translation into interpretable exploratory phenotype-translation analyses.

The results from this study should be interpreted considering multiple limitations. The study is single-centre and ambispective, and external validation in independent, ideally multi-ethnic populations is essential before clinical adoption. Outcome ascertainment was incomplete for a proportion of the cohort, although inverse-probability weighting suggested minimal impact. Rare events such as early preterm birth and low Apgar were represented by small numbers, producing wide confidence intervals. Finally, phenotypes derived from unsupervised clustering are exploratory by design. The associations reported here are hypothesis-generating and require prospective confirmation before the phenotype can be recommended for clinical decision-making. In addition, the modest sensitivity of Phenotype 4 and incomplete outcome ascertainment limit its immediate applicability for clinical screening and reinforce the exploratory nature of the present findings.

Prospective multicentre validation is the immediate next step, ideally including comparison with established first-trimester prediction models such as the Fetal Medicine Foundation competing-risks algorithm. Future studies should determine whether AI-derived phenotypes provide incremental information beyond existing prediction models and whether they can be reproduced across different populations. Only after prospective external validation should their potential role in risk stratification or individualized surveillance be evaluated.

Longitudinal follow-up beyond delivery, including postpartum cardiovascular and metabolic outcomes, would test whether Phenotype 4 also marks women at elevated long-term cardiometabolic risk, consistent with its early-pregnancy signature. Incorporation of additional biomarkers, particularly placental growth factor, angiogenic imbalance, and metabolomic signatures, may further refine the phenotype and clarify its biological significance.

An additional potential application of unsupervised phenotyping may lie in clinical settings where robust prediction models have not yet been established. Rather than competing with well-validated outcome-specific screening algorithms, clustering could be used as an exploratory tool to identify recurrent combinations of clinical, biophysical, biochemical, or imaging variables that may subsequently inform the development of new supervised prediction models.

Also, further studies could expand the input data beyond variables already incorporated into established obstetric risk models. In the present study, several of the characteristics contributing to the high-risk phenotype are established markers of adverse pregnancy outcomes. Consequently, the emergence of a cardiometabolic–vascular high-risk phenotype from these variables is biologically and clinically plausible, but not entirely unexpected. Future unsupervised analyses may be more informative if they integrate less conventionally modeled dimensions of pregnancy risk, including socioeconomic deprivation, educational level, health literacy, access to antenatal care, geographic barriers to healthcare, environmental exposures, nutritional factors, psychosocial characteristics, and longitudinal patterns of healthcare utilization. Combining these contextual determinants with conventional maternal, biophysical, biochemical, and imaging data could reveal risk phenotypes that are not readily captured by existing outcome-specific screening algorithms. Such an approach may be particularly valuable for hypothesis generation and for identifying candidate variables, interactions, and patient subgroups that could subsequently be evaluated in dedicated, prospectively validated prediction models.

## 5. Conclusions

Unsupervised machine learning applied to routine first-trimester variables identified a small, reproducible maternal cardiometabolic–vascular phenotype that was associated with enrichment for multiple adverse maternal and perinatal outcomes. The phenotype remained identifiable across alternative clustering algorithms and multiple sensitivity analyses, supporting the internal robustness of the observed clustering structure.

Exploratory phenotype-translation analyses suggested that the AI-derived phenotype could be approximated using simpler first-trimester clinical variables; however, these analyses reproduced phenotype membership rather than obstetric outcomes and should not be interpreted as clinical prediction models. Overall, the present findings should be regarded as hypothesis-generating and require prospective multicentre external validation before any role in clinical risk stratification, surveillance, or decision-making can be considered.

## Figures and Tables

**Table 1:** Comparison of maternal characteristics and first-trimester biomarkers across the four phenotypes.

Characteristic	Phenotype 1 (*n* = 281)	Phenotype 2 (*n* = 837)	Phenotype 3 (*n* = 405)	Phenotype 4 (*n* = 60)	*p* Value
Age (years)	33.00 (29.90–36.20)	30.00 (27.20–32.80)	28.90 (26.40–32.30)	34.40 (30.05–38.15)	<0.001
BMI (kg/m^2^)	23.44 (21.23–26.49)	22.04 (20.31–24.61)	22.04 (20.20–24.38)	28.19 (23.86–32.33)	<0.001
Gravidity	2 (2–3)	1 (1–2)	1 (1–1)	2 (1–3)	<0.001
Parity	1 (1–1)	0 (0–0)	0 (0–0)	0.5 (0–1)	<0.001
Obstetric history	99.3% parous, no previous PE	100% nulliparous	100% nulliparous	50.0% nulliparous; 45.0% previous PE; 5.0% parous, no previous PE	<0.001
Conception	98.9% spontaneous	89.0% spontaneous; 6.5% IVF	96.5% spontaneous	83.3% spontaneous; 13.3% IVF	<0.001
Smoking during pregnancy	10.3%	9.0%	6.7%	5.0%	0.271
Diabetes mellitus	1.1% diabetes	0.1% diabetes	1.7% diabetes	1.7% diabetes	0.026
Chronic hypertension	0%	0%	0%	56.7%	<0.001
Maternal family history of PE	0.7%	0.7%	0%	1.7%	0.254
MAP (mmHg)	89.83 (84.40–93.08)	89.18 (84.65–93.33)	87.00 (82.45–91.68)	101.00 (95.29–107.12)	<0.001
UtA-PI	1.59 (1.33–1.96)	1.63 (1.35–1.96)	1.67 (1.39–2.02)	1.48 (1.31–1.87)	0.030
PAPP-A (MoM)	1.05 (0.64–1.50)	0.98 (0.66–1.46)	0.75 (0.43–1.05)	0.96 (0.75–1.33)	<0.001
NT (MoM)	1.70 (1.40–2.00)	1.60 (1.40–1.80)	1.90 (1.70–2.20)	1.70 (1.50–1.90)	<0.001

Legend: BMI—body mass index; PE—preeclampsia; IVF—in vitro fertilization; MAP—mean arterial pressure; UtA-PI—mean uterine artery pulsatility index; PAPP-A—pregnancy-associated plasma protein A; NT—nuchal translucency; IQR—interquartile range. Continuous variables are presented as median (IQR) and categorical variables as *n* (%). Descriptive statistics were calculated from the observed (non-imputed) dataset. The observed sample size for each variable is reported where missing data were present. Median imputation was used exclusively for PCA and clustering analyses.

**Table 2:** Pregnancy and neonatal outcomes according to the four identified phenotypes.

Outcome	Phenotype 1	Phenotype 2	Phenotype 3	Phenotype 4	*p* Value
Gestational hypertension	1/94 (1.1%)	28/509 (5.5%)	19/241 (7.9%)	12/42 (28.6%)	<0.001
Gestational diabetes mellitus	16/94 (17.0%)	36/509 (7.1%)	11/241 (4.6%)	14/42 (33.3%)	<0.001
Preterm birth (<37 weeks)	3/77 (3.9%)	39/478 (8.2%)	21/232 (9.1%)	6/37 (16.2%)	0.154
Early preterm birth (<34 weeks)	2/77 (2.6%)	11/478 (2.3%)	10/232 (4.3%)	4/37 (10.8%)	0.034
Emergency cesarean section	13/78 (16.7%)	54/479 (11.3%)	25/232 (10.8%)	10/37 (27.0%)	0.020
1-min Apgar score < 7	0/67 (0.0%)	8/441 (1.8%)	12/216 (5.6%)	3/37 (8.1%)	0.009
Low birth weight (<2500 g)	3/75 (4.0%)	23/471 (4.9%)	15/232 (6.5%)	4/37 (10.8%)	0.388
Small for gestational age (<10th percentile)	7/70 (10.0%)	58/436 (13.3%)	25/207 (12.1%)	2/31 (6.5%)	0.631
Maternal metabolic or hypertensive complications	17/94 (18.1%)	60/509 (11.8%)	25/241 (10.4%)	22/42 (52.4%)	<0.001
Delivery or neonatal compromise	14/78 (17.9%)	68/483 (14.1%)	40/234 (17.1%)	12/37 (32.4%)	0.029
Clinically actionable adverse pregnancy outcome	30/94 (31.9%)	120/509 (23.6%)	58/241 (24.1%)	24/42 (57.1%)	<0.001
Expanded clinically actionable adverse obstetric outcome	31/94 (33.0%)	141/509 (27.7%)	66/241 (27.4%)	25/42 (59.5%)	<0.001
Mean weighted adverse burden score	0.39	0.35	0.47	1.29	<0.001
Mean adverse event count	0.37	0.31	0.38	1.12	<0.001
Burden score ≥ 2	4.3%	6.9%	12.4%	28.6%	<0.001
Burden score ≥ 3	2.1%	1.8%	4.6%	9.5%	0.014

Legend: GDM—gestational diabetes mellitus; CS—cesarean section; APO—adverse pregnancy outcome; SGA—small for gestational age.

**Table 3:** Relative risk of adverse maternal and perinatal outcomes in Phenotype 4 compared with the remaining phenotypes.

Outcome	Phenotype 4 (*n*)	Events	Event Rate	Relative Risk (RR)	95% CI	*p* Value	FDR *q* Value	E-Value	E-Value (95% CI Limit)
Maternal metabolic or hypertensive complications	42	22	52.4%	4.314	3.076–6.049	<0.001	<0.001	8.094	5.603
Clinically actionable adverse pregnancy outcomes	42	24	57.1%	2.309	1.736–3.071	<0.001	<0.001	4.048	2.867
Expanded clinically actionable adverse pregnancy outcome	42	25	59.5%	2.101	1.604–2.752	<0.001	<0.001	3.622	2.588
Delivery or neonatal compromise	37	12	32.4%	2.137	1.319–3.463	0.029	0.037	3.697	1.969
Gestational hypertension	42	12	28.6%	5.065	2.949–8.700	<0.001	<0.001	9.602	5.346
Gestational diabetes mellitus	42	14	33.3%	4.487	2.773–7.260	<0.001	<0.001	8.443	4.991
Early preterm birth (<34 weeks)	37	4	10.8%	3.971	1.529–10.315	0.035	0.039	7.406	2.428
Emergency cesarean section	37	10	27.0%	2.360	1.363–4.086	0.022	0.031	4.151	2.066
1-min Apgar score < 7	37	3	8.1%	3.257	1.100–9.646	0.012	0.020	5.969	1.432

Legend: RR—relative risk; CI—confidence interval; FDR—false discovery rate; APO—adverse pregnancy outcome; CS—cesarean section.

**Table 4:** Predictive performance of Phenotype 4 for adverse maternal and perinatal outcomes.

Outcome	*N*	Events	Phenotype 4 Flagged (*n*)	Events in Phenotype 4	Sensitivity	Specificity	PPV	NPV	LR+	LR−	**Risk in Phenotype 4**	**Risk in Other Phenotypes**	**Absolute Risk Difference**
Maternal metabolic/hypertensive complications	886	124	42	22	0.177	0.974	0.524	0.879	6.760	0.845	0.524	0.121	0.403
Clinically actionable APO	886	232	42	24	0.103	0.972	0.571	0.754	3.759	0.922	0.571	0.246	0.325
Expanded clinically actionable APO	886	263	42	25	0.095	0.973	0.595	0.718	3.484	0.930	0.595	0.282	0.313
Delivery or neonatal compromise	832	134	37	12	0.090	0.964	0.324	0.847	2.500	0.944	0.324	0.153	0.171
Early preterm birth (<34 weeks)	824	27	37	4	0.148	0.959	0.108	0.971	3.578	0.889	0.108	0.029	0.079
Emergency cesarean section	826	102	37	10	0.098	0.963	0.270	0.883	2.629	0.937	0.270	0.117	0.154
1-min Apgar score < 7	761	23	37	3	0.130	0.954	0.081	0.972	2.831	0.912	0.081	0.028	0.053

Legend: APO—adverse pregnancy outcome; PPV—positive predictive value; NPV—negative predictive value; LR+—positive likelihood ratio; LR−—negative likelihood ratio.

**Table 5:** Cross-algorithm agreement, Phenotype 4 recovery, and phenotype translation.

Global Cross-Algorithm Agreement						
Method	Number of clusters		ARI		AMI	
Diagonal Gaussian mixture model	4		0.763		0.770	
Two-stage Ward hierarchical clustering	4		0.660		0.695	
Phenotype 4-specific recovery						
Method	Matched cluster size	Primary Phenotype 4 overlap	Sensitivity	Specificity	PPV	Jaccard similarity
Diagonal Gaussian mixture model	71	60/60	1.000	0.993	0.845	0.845
Two-stage Ward hierarchical clustering	59	56/60	0.933	0.998	0.949	0.889
Contingency table: primary PCA + k-means vs. diagonal Gaussian mixture model						
Primary phenotype	GMM Phenotype 1	GMM Phenotype 2	GMM Phenotype 3		GMM Phenotype 4	Row total
Phenotype 1	280	0	0		1	281
Phenotype 2	0	817	14		6	837
Phenotype 3	0	107	294		4	405
Phenotype 4	0	0	0		60	60
Column total	280	924	308		71	1583
Primary PCA + k-means vs. two-stage Ward hierarchical clustering						
Primary phenotype	Ward Phenotype 1	Ward Phenotype 2	Ward Phenotype 3		Ward Phenotype 4	Row total
Phenotype 1	278	0	0		3	281
Phenotype 2	0	722	115		0	837
Phenotype 3	0	69	336		0	405
Phenotype 4	1	3	0		56	60
Column total	279	794	451		59	1583
Phenotype translation						
Method	Sensitivity		Specificity		PPV	AUROC
Cross-validated logistic surrogate model	0.617		0.983		0.587	0.887 (0.823–0.940
Simple points-based score (≥3)	0.617		0.991		0.725	0.884 (0.828–0.931)

Legend: ARI—adjusted Rand index; AMI—adjusted mutual information; PPV—positive predictive value; AUROC—area under the receiver operating characteristic curve; PCA—principal component analysis; GMM—Gaussian mixture model.

## Data Availability

The datasets are available from the correspondent author upon a reasonable request due to local policies.

## Supplementary Materials

The following supporting information can be downloaded at: https://www.mdpi.com/article/10.3390/diagnostics16162654/s1, Table S1: PCA dimensionality reduction and k-means implementation; Table S2: Cluster-number selection across candidate k values; Table S3: Fixed-k stability analysis across candidate cluster solutions; Table S4: Contribution of individual variables to the retained principal components; Table S5: Highest PCA loadings for retained principal components; Table S6: Outcome availability overall and according to AI-derived phenotype; Table S7: Source variables and endpoint-specific denominators; Table S8a: Comparison of pregnancies with and without documented principal maternal outcome data; Table S8b: Specification and diagnostics of the inverse probability weighting model; Table S9: Covariate balance before and after inverse probability weighting; Table S10: Complete-case and inverse probability weighted estimates for Phenotype 4; Table S11: Missingness of variables used for phenotype derivation; Table S12: Sensitivity analyses evaluating preprocessing strategies; Table S13: Mixed-data clustering sensitivity analyses; Table S14: Comparison of patients with and without chronic hypertension within Phenotype 4; Table S15: Baseline comparison between the retained four-cluster solution (k = 4) and the alternative five-cluster solution (k = 5); Table S16: Sensitivity analyses; Table S17: Comparison of observed and inverse probability weighted (IPW) outcome rates among patients classified as Phenotype 4; Table S18: Leakage-safe internal phenotype-replication performance and calibration of the Phenotype 4 translation models; Table S19: Complete specification of the simple points-based score for approximation of Phenotype 4 membership; Table S20: Specification of the logistic surrogate model used for Phenotype 4 translation; Table S21: Cross-validation structure and threshold selection for the Phenotype 4 translation analyses; Table S22: Incremental predictive performance of AI-derived phenotype membership; Table S23: Bootstrap validation of incremental predictive performance after addition of AI-derived phenotype membership.
