# Supplementary material for: Unsupervised Machine Learning for the Identification of Latent First-Trimester Obstetric Phenotypes Associated with Maternal and Perinatal Morbidity
Source: Diagnostics (Basel). 2026 Aug 20;16(16):2654. doi: 10.3390/diagnostics16162654 (PMC13523598; doi:10.3390/diagnostics16162654)
Supplement: Supplementary file 1 [file diagnostics-16-02654-s001.zip › diagnostics-4469077-supplementary.pdf]

Supplementary Table S1. PCA dimensionality reduction and k-means implementation

Parameter	Value
Cohort used for clustering	1,583 pregnancies
Original clinical variables	23
Encoded features entering PCA	39
Retained principal components	4
PC1 variance explained	14.3%
PC2 variance explained	9.4%
PC3 variance explained	7.6%
PC4 variance explained	6.7%
**Cumulative explained variance**	**38.1%**
Clustering space	First four principal-component scores
Candidate k values	2–6
Initialization	k-means++
Number of random starts (n_init)	30
Maximum iterations	300
Distance metric	Euclidean
Random seed	20260708
Stability assessment	Fixed-*k* refitting using identical 200 random 80% subsamples
Outcome variables used during cluster selection	No

Legend. This table summarizes the dimensionality-reduction procedure and k-means implementation used in the primary phenotype-discovery pipeline.

Supplementary Table S2. Cluster-number selection across candidate k values

k	Inertia (WCSS)	Mean silhouette	Calinski–Harabasz	Davies–Bouldin	Median fixed-*k* ARI	Fixed-*k* ARI (IQR)	Cluster sizes	Interpretation
2	15064.0	**0.467**	**884.1**	**0.956**	**1.000**	1.000–1.000	311; 1272	Strongest geometric separation but clinically coarse partition.
3	11911.7	0.323	767.8	1.243	0.933	0.874–0.958	435; 311; 837	Stable intermediate solution without preserving all clinically distinct baseline profiles.
**4**	**9823.2**	**0.354**	**732.2**	**1.112**	**0.891**	**0.532–0.948**	**405; 281; 60; 837**	**Selected solution balancing statistical adequacy, cluster-size plausibility, stability, and clinically interpretable baseline phenotypes.**
5	7812.8	0.345	791.5	0.995	0.945	0.919–0.956	446; 158; 59; 281; 639	Higher statistical stability but fragmentation of existing phenotypes rather than emergence of a novel clinical subgroup.
6	7058.1	0.301	734.2	1.074	0.852	0.633–0.936	389; 152; 405; 58; 280; 299	Greater fragmentation with lower stability.

Legend. Candidate PCA+k-means solutions (k=2–6) were evaluated using within-cluster sum of squares (WCSS), mean silhouette coefficient, Calinski–Harabasz index, Davies–Bouldin index, fixed-*k* subsampling stability, cluster-size distribution, and outcome-independent clinical interpretability. Outcome variables were not used during cluster-number selection.

Supplementary Table S3. Fixed-*k* stability analysis across candidate cluster solutions

k	Median ARI	Q1	Q3	IQR	5th percentile	95th percentile	Minimum	Maximum
2	1.000	1.000	1.000	1.000–1.000	1.000	1.000	1.000	1.000
3	0.933	0.874	0.958	0.874–0.958	0.822	0.982	0.784	0.992
**4**	**0.891**	**0.532**	**0.948**	**0.532–0.948**	**0.507**	**0.976**	**0.497**	**0.988**
5	0.945	0.919	0.956	0.919–0.956	0.890	0.974	0.852	0.990
6	0.852	0.633	0.936	0.633–0.936	0.605	0.955	0.590	0.967

Legend. Stability was evaluated by repeating the complete preprocessing, PCA, and k-means procedure within the same 200 random 80% subsamples while fixing the candidate value of *k*. Stability was quantified by the Adjusted Rand Index (ARI) between the refitted subsample solution and the corresponding full-cohort solution restricted to the same observations.

Supplementary Table S4. Contribution of individual variables to the retained principal components

Principal Component	Main contributing variables (highest absolute loadings)	Chronic hypertension contribution
**PC1 (14.3%)**	Nulliparity, parity history, gravidity, maternal age, BMI	Rank **22**
**PC2 (9.4%)**	Gestational age, CRL, NT, missing biochemical markers	Rank **15**
**PC3 (7.6%)**	MAP, **chronic hypertension**, BMI, weight, previous preeclampsia	Rank **2–3**
**PC4 (6.7%)**	Missing MAP/PAPP-A, uterine artery PI, CRL	Rank **17**
**PC5 (5.7%)**	UtA-PI, PAPP-A, diabetes, smoking	Rank **9–10**
**PC6 (5.4%)**	Family history of preeclampsia, previous preeclampsia, conception method	Rank **37**

Abbreviations: BMI, body mass index; MAP, mean arterial pressure; CRL, crown-rump length; NT, nuchal translucency; UtA-PI, uterine artery pulsatility index.

Supplementary Table S5. Highest PCA loadings for retained principal components

PC	Explained variance (%)	Highest positive loadings	Highest negative loadings
**PC1**	14.3	Parity, previous PE history, gravidity	Nulliparity
**PC2**	9.4	Gestational age, CRL, NT	PAPP-A, MAP
**PC3**	7.6	MAP, chronic hypertension, BMI, weight	UtA-PI
**PC4**	6.7	Missing biochemical markers	CRL, gestational age
**PC5**	5.7	PAPP-A, diabetes	UtA-PI, smoking
**PC6**	5.4	Previous PE, conception method	Family history variables

Supplementary Table S6. Outcome availability overall and according to AI-derived phenotype

Outcome	Overall available, n/N (%)	Phenotype 1	Phenotype 2	Phenotype 3	Phenotype 4
Maternal metabolic or hypertensive complications	886/1,583 (56.0)	94/281 (33.5)	509/837 (60.8)	241/405 (59.5)	42/60 (70.0)
Clinically actionable obstetric adverse event	886/1,583 (56.0)	94/281 (33.5)	509/837 (60.8)	241/405 (59.5)	42/60 (70.0)
Broad clinically actionable APO	886/1,583 (56.0)	94/281 (33.5)	509/837 (60.8)	241/405 (59.5)	42/60 (70.0)
Delivery or neonatal compromise	832/1,583 (52.6)	78/281 (27.8)	483/837 (57.7)	234/405 (57.8)	37/60 (61.7)

Gestational hypertension	886/1,583 (56.0)	94/281 (33.5)	509/837 (60.8)	241/405 (59.5)	42/60 (70.0)
Gestational diabetes mellitus	886/1,583 (56.0)	94/281 (33.5)	509/837 (60.8)	241/405 (59.5)	42/60 (70.0)
Preterm birth (<37 weeks)	824/1,583 (52.1)	77/281 (27.4)	478/837 (57.1)	232/405 (57.3)	37/60 (61.7)
Early preterm birth (<34 weeks)	824/1,583 (52.1)	77/281 (27.4)	478/837 (57.1)	232/405 (57.3)	37/60 (61.7)
Emergency cesarean section	826/1,583 (52.2)	78/281 (27.8)	479/837 (57.2)	232/405 (57.3)	37/60 (61.7)
1-minute Apgar score <7	761/1,583 (48.1)	67/281 (23.8)	441/837 (52.7)	216/405 (53.3)	37/60 (61.7)
Low birth weight (<2500 g)	815/1,583 (51.5)	75/281 (26.7)	471/837 (56.3)	232/405 (57.3)	37/60 (61.7)
SGA proxy (<10th percentile)	744/1,583 (47.0)	70/281 (24.9)	436/837 (52.1)	207/405 (51.1)	31/60 (51.7)
Composite adverse fetal outcome	886/1,583 (56.0)	94/281 (33.5)	509/837 (60.8)	241/405 (59.5)	42/60 (70.0)

Legend. Availability of maternal, obstetric, delivery, and neonatal outcomes in the overall cohort and within each AI-derived phenotype. Percentages refer to the proportion of patients with sufficient information to derive the corresponding endpoint and should not be interpreted as event rates.
Abbreviations: AI – artificial intelligence; APO – adverse pregnancy outcome; SGA – small for gestational age.

Supplementary Table S7. Source variables and endpoint-specific denominators

Endpoint	Source variables or derivation rule	Available N (%)	Phenotype 1	Phenotype 2	Phenotype 3	Phenotype 4
Maternal metabolic or hypertensive complications	Ge_hta, Gest_dz, Diab_gest; non-events defined only when outcome documentation was present	886 (56.0)	94	509	241	42
Clinically actionable obstetric adverse event	Ge_hta, Gest_dz/Diab_gest, gestational age at delivery, Nast_tip, Apgar1	886 (56.0)	94	509	241	42
Delivery or neonatal compromise	Gestational age at delivery, Nast_tip, Apgar1	832 (52.6)	78	483	234	37
Preterm birth <37 weeks	del_ga_wk, del_ga_d	824 (52.1)	77	478	232	37
Early preterm birth <34 weeks	del_ga_wk, del_ga_d	824 (52.1)	77	478	232	37
Emergency cesarean section	Nast_tip	826 (52.2)	78	479	232	37
Low 1-minute Apgar score	Apgar1	761 (48.1)	67	441	216	37
Low birth weight	Greut_nast	815 (51.5)	75	471	232	37
SGA proxy	Sex, gestational age at delivery, birth weight, and stratum size ≥10	744 (47.0)	70	436	207	31

Legend. Endpoint-specific source variables and resulting denominators used in post hoc outcome analyses. Differences in denominators reflect availability of the source fields required to derive each maternal, delivery, or neonatal outcome.
Abbreviations: SGA – small for gestational age.

Supplementary Table S8a. Comparison of pregnancies with and without documented principal maternal outcome data

Characteristic	Documented	Not documented	Statistical test	*p* value	Standardized difference
Age, years	30.00 (27.20–33.10)	30.80 (28.00–34.20)	Permutation median-difference test	0.0017	−0.155
BMI, kg/m²	22.29 (20.33–25.45)	22.41 (20.55–25.00)	Permutation median-difference test	0.6491	0.032
Gravidity	1.00 (1.00–2.00)	1.00 (1.00–2.00)	Permutation median-difference test	1.000	−0.183
Parity	0.00 (0.00–0.00)	0.00 (0.00–1.00)	Permutation median-difference test	1.000	−0.369
MAP, mmHg	89.00 (84.41–93.90)	89.42 (84.40–92.58)	Permutation median-difference test	0.3759	0.135
Mean UtA-PI	1.64 (1.37–1.97)	1.60 (1.32–1.99)	Permutation median-difference test	0.2303	0.037
PAPP-A, MoM	0.92 (0.59–1.34)	1.01 (0.65–1.51)	Permutation median-difference test	0.0403	−0.156
Nuchal translucency	1.70 (1.40–1.90)	1.70 (1.40–1.90)	Permutation median-difference test	1.000	−0.019
Conception method	IVF: 33/886 (3.7%); ovulation drugs: 33/886 (3.7%); spontaneous: 820/886 (92.6%)	IVF: 41/697 (5.9%); ovulation drugs: 12/697 (1.7%); spontaneous: 644/697 (92.4%)	χ^2 test	0.0092	0.123
Smoking	No: 822/886 (92.8%); Yes: 64/886 (7.2%)	No: 627/697 (90.0%); Yes: 70/697 (10.0%)	χ^2 test	0.0429	−0.101
Pre-existing diabetes mellitus	No: 880/886 (99.3%); Type 1: 4/886 (0.5%); Type 2: 2/886 (0.2%)	No: 691/697 (99.1%); Type 1: 5/697 (0.7%); Type 2: 1/697 (0.1%)	χ^2 test	0.7357	−0.035
Chronic hypertension	No: 860/886 (97.1%); Yes: 26/886 (2.9%)	No: 689/697 (98.9%); Yes: 8/697 (1.1%)	χ^2 test	0.0143	−0.127
Previous preeclampsia	No: 868/886 (98.0%); Yes: 18/886 (2.0%)	No: 686/697 (98.4%); Yes: 11/697 (1.6%)	χ^2 test	0.5117	−0.034
Phenotype distribution	P1: 94 (10.6%); P2: 509 (57.4%); P3: 241 (27.2%); P4: 42 (4.7%)	P1: 187 (26.8%); P2: 328 (47.1%); P3: 164 (23.5%); P4: 18 (2.6%)	χ^2 test	<0.001	−0.425

Legend. Comparison of baseline characteristics between pregnancies with and without documentation of the principal maternal metabolic/hypertensive outcome. Continuous variables are presented as median (interquartile range) and categorical variables as n (%). Standardized differences are provided to quantify the magnitude of between-group imbalance independently of sample size.

Abbreviations: BMI – body mass index; MAP – mean arterial pressure; UtA-PI – uterine artery pulsatility index; PAPP-A – pregnancy-associated plasma protein A; MoM – multiples of the median; IVF – in vitro fertilization.

Supplementary Table S8b. Specification and diagnostics of the inverse probability weighting model

Characteristic	Value
Documentation indicator	Maternal metabolic or hypertensive outcome available
Documented pregnancies	886
Non-documented pregnancies	697
Model	L2-penalized logistic regression
Penalization	L2 penalty = 1.0
Continuous covariate processing	Standardized within design matrix
Covariates	Age, BMI, gravidity, gravidity missingness indicator, parity, conception method, smoking, pre-existing diabetes mellitus, chronic hypertension, and phenotype indicators
Previous preeclampsia included	No
Stabilized weights	Yes
Propensity-score clipping	0.05–0.99
Additional weight truncation	None
Minimum weight	0.660
1st percentile	0.754
5th percentile	0.822
Median weight	1.012
Mean weight	1.117
95th percentile	1.921
99th percentile	2.412
Maximum weight	3.488
Effective sample size	811.1
Minimum raw predicted probability	0.0047
Maximum raw predicted probability	0.8482

Legend. Specification and diagnostic characteristics of the inverse probability weighting model used to account for differential documentation of the principal maternal outcome. Stabilized weights were calculated after clipping predicted documentation probabilities to 0.05–0.99; no additional weight truncation was applied.
Abbreviations: IPW – inverse probability weighting; BMI – body mass index.

Supplementary Table S9. Covariate balance before and after inverse probability weighting

Covariate	Unweighted standardized difference	Weighted standardized difference	Absolute unweighted difference	Absolute weighted difference
Age	−0.155	0.001	0.155	0.001
BMI	0.032	−0.008	0.032	0.008
Gravidity	−0.182	−0.003	0.182	0.003
Gravidity missingness indicator	−0.054	−0.039	0.054	0.039

Parity	−0.369	−0.001	0.369	0.001
Ovulation induction conception	0.123	−0.001	0.123	0.001
Spontaneous conception	0.006	0.004	0.006	0.004
Smoking during pregnancy	−0.101	−0.002	0.101	0.002
Type 1 diabetes mellitus	−0.035	0.017	0.035	0.017
Type 2 diabetes mellitus	0.019	0.005	0.019	0.005
Chronic hypertension	0.127	−0.009	0.127	0.009
Phenotype 1	−0.425	0.001	0.425	0.001
Phenotype 3	0.085	0.002	0.085	0.002
Phenotype 4	0.115	−0.011	0.115	0.011

Legend. Standardized differences between pregnancies with and without documented principal maternal outcomes before and after inverse probability weighting. Weighting markedly reduced measured imbalance, with the maximum absolute standardized difference decreasing from 0.425 to 0.039.
Abbreviations: BMI – body mass index; IPW – inverse probability weighting.

Supplementary Table S10. Complete-case and inverse probability weighted estimates for Phenotype 4

Outcome	Phenotype 4 complete-case	Other phenotypes complete-case	Complete-case RR (95% CI)	Phenotype 4 IPW rate	Other phenotypes IPW rate	IPW RR	Robust 95% CI	Bootstrap 95% CI
Maternal metabolic or hypertensive complications	22/42 (52.4%)	102/844 (12.1%)	4.31 (3.08–6.05)	51.1%	12.8%	3.99	2.79–5.70	2.66–5.59
Clinically actionable obstetric adverse event	24/42 (57.1%)	208/844 (24.6%)	2.31 (1.74–3.07)	55.1%	25.6%	2.15	1.59–2.92	1.56–2.82
Delivery or neonatal compromise	12/37 (32.4%)	122/795 (15.3%)	2.14 (1.32–3.46)	30.2%	15.7%	1.92	1.15–3.23	1.00–3.01
Early preterm birth (<34 weeks)	4/37 (10.8%)	23/787 (2.9%)	3.97 (1.53–10.31)	8.9%	2.8%	3.18	1.13–8.96	0.62–7.76
Emergency cesarean section	10/37 (27.0%)	92/789 (11.7%)	2.36 (1.36–4.09)	26.0%	12.3%	2.11	1.18–3.77	0.98–3.61
1-minute Apgar score <7	3/37 (8.1%)	20/724 (2.8%)	3.26 (1.10–9.65)	6.5%	2.5%	2.61	0.79–8.56	0.00–7.12

Legend. Side-by-side comparison of complete-case and inverse probability weighted estimates for adverse outcomes in Phenotype 4 compared with the remaining phenotypes. Robust confidence intervals were calculated using sandwich-type standard errors based on the influence function for weighted risk ratios. Bootstrap confidence intervals were derived from 2,000 nonparametric bootstrap repetitions.
Abbreviations: RR – relative risk; CI – confidence interval; IPW – inverse probability weighting.

Supplementary Table S11. Missingness of variables used for phenotype derivation

Variable	Type	Total N	Missing, n	Missing, %
Age	Continuous	1,583	0	0.00
Gravidity	Continuous	1,583	1	0.06
Parity	Continuous	1,583	0	0.00
Maternal weight	Continuous	1,583	0	0.00
Maternal height	Continuous	1,583	0	0.00
BMI	Continuous	1,583	0	0.00
Crown–rump length	Continuous	1,583	0	0.00
Nuchal translucency	Continuous	1,583	9	0.57
Gestational age at examination, days	Continuous	1,583	0	0.00
Gestational age at examination, weeks	Continuous	1,583	0	0.00
Mean arterial pressure	Continuous	1,583	180	11.37
MAP-derived variable (m1)	Continuous	1,583	180	11.37
Mean uterine artery PI	Continuous	1,583	0	0.00
UtA-PI-derived variable (u1)	Continuous	1,583	0	0.00
PAPP-A	Continuous	1,583	479	30.26
Log-transformed PAPP-A	Continuous	1,583	479	30.26
Previous obstetric history	Categorical	1,583	0	0.00
Conception method	Categorical	1,583	0	0.00
Smoking during pregnancy	Categorical	1,583	0	0.00
Pre-existing diabetes mellitus	Categorical	1,583	0	0.00
Chronic hypertension	Categorical	1,583	0	0.00
Maternal family history of preeclampsia	Categorical	1,583	0	0.00
Parity category	Categorical	1,583	0	0.00

Legend. Missingness of variables included in the final phenotype derivation pipeline. Continuous variables with missing values were handled according to the predefined preprocessing strategy before clustering.
Abbreviations: BMI – body mass index; MAP – mean arterial pressure; PI – pulsatility index; UtA-PI – uterine artery pulsatility index; PAPP-A – pregnancy-associated plasma protein A.

Supplementary Table S12. Sensitivity analyses evaluating preprocessing strategies

Analysis	Matched cluster size	P4 overlap	Sensitivity	Specificity	PPV	Jaccard	Global ARI	Global NMI
No hypertensive-history variables	447	47/60	0.783	0.737	0.105	0.102	0.196	0.242
Binary variables retained as 0/1	288	40/60	0.667	0.837	0.139	0.130	0.188	0.216
Binary variables down-weighted	287	44/60	0.733	0.840	0.153	0.145	0.142	0.160
Reference coding (k−1 dummies)	281	42/60	0.700	0.843	0.149	0.140	0.150	0.170

Supplementary Table S13. Mixed-data clustering sensitivity analyses

Method	Cluster size	P4 overlap	Sensitivity	Specificity	PPV	Jaccard	Global ARI	Global NMI
Gower/PAM (forced *k*=4)	19	11/60	0.183	0.995	0.579	0.162	0.137	0.132
Gower/PAM (selected *k*=2)	306	26/60	0.433	0.816	0.085	0.076	0.428	0.622

Supplementary Table S14. Comparison of patients with and without chronic hypertension within Phenotype 4

Characteristic	P4 without chronic hypertension (n=26)	P4 with chronic hypertension (n=34)
BMI, median	28.27	28.03
Mean arterial pressure, median	94.5	105.9
Previous preeclampsia (%)	84.6	14.7
Gestational hypertension/Superimposed PE	3/16 (18.8%)	9/26 (34.6%)
Gestational diabetes	5/16 (31.2%)	9/26 (34.6%)

Supplementary Table S15. Baseline comparison between the retained four-cluster solution (k = 4) and the alternative five-cluster solution (k = 5)

k=5 cluster	n	Corresponding retained phenotype(s)	Age (years)	BMI (kg/m²)	NT (mm)	MAP (mmHg)	Mean UtA-PI	PAPP-A MoM
**K5-C4**	281	Phenotype 1 (281/281)	33.00 (29.90–36.20)	23.44 (21.23–26.49)	1.70 (1.40–2.00)	89.83 (84.40–93.08)	1.59 (1.33–1.96)	1.05 (0.64–1.50)
**K5-C5**	639	Phenotype 2 (620) + Phenotype 3 (19)	30.10 (27.30–32.80)	21.97 (20.31–24.52)	1.50 (1.30–1.70)	89.33 (84.71–93.40)	1.67 (1.40–2.01)	0.98 (0.66–1.55)
**K5-C1**	446	Phenotype 2 (217) + Phenotype 3 (228)	29.60 (27.10–32.88)	22.05 (20.20–24.79)	1.90 (1.70–2.20)	88.00 (83.17–92.42)	1.55 (1.27–1.87)	0.89 (0.56–1.25)
**K5-C2**	158	Phenotype 3 (158/158)	28.10 (26.02–31.20)	22.05 (20.57–24.62)	1.70 (1.48–1.90)	90.84 (86.30–93.70)	1.83 (1.51–2.22)	0.68 (0.27–1.06)
**K5-C3**	59	Phenotype 4 (59/60)	34.50 (30.00–38.20)	28.06 (23.84–32.16)	1.65 (1.50–1.90)	101.00 (94.90–107.44)	1.50 (1.32–1.88)	0.96 (0.75–1.33)

Legend. Comparison of the retained four-cluster solution with the alternative five-cluster solution demonstrated that increasing *k* did not identify an additional clinically distinct phenotype. Instead, the fifth cluster primarily fragmented the original nulliparous phenotypes (Phenotypes 2 and 3), whereas Phenotypes 1 and 4 were almost completely preserved (281/281 and 59/60 patients, respectively). This finding supported retention of the four-cluster solution as the most parsimonious clinically interpretable representation of the data.

Supplementary Table S16. Sensitivity analyses

Population	Outcome	RR	95% CI	*p* value
Nulliparous only	Maternal metabolic or hypertensive complications	4.743	3.138–7.170	<0.001
	Delivery or neonatal compromise	3.309	2.123–5.158	<0.001
	Clinically actionable obstetric adverse event	2.609	1.871–3.638	<0.001
	Early preterm birth (<34 weeks)	6.470	2.560–16.355	0.002
	Emergency cesarean section	3.699	2.179–6.281	<0.001
Spontaneous conception only	Maternal metabolic or hypertensive complications	4.482	3.066–6.551	<0.001
	Delivery or neonatal compromise	2.008	1.157–3.484	0.105
	Clinically actionable obstetric adverse event	2.324	1.697–3.182	<0.001
	Early preterm birth (<34 weeks)	3.999	1.373–11.649	0.047
	Emergency cesarean section	2.346	1.286–4.280	0.042

PAPP-A available only	Maternal metabolic or hypertensive complications	3.830	2.550–5.752	<0.001
	Delivery or neonatal compromise	2.127	1.205–3.755	0.008
	Clinically actionable obstetric adverse event	2.230	1.588–3.132	<0.001
	Early preterm birth (<34 weeks)	3.439	0.929–12.729	0.378
	Emergency cesarean section	2.347	1.265–4.356	0.003

Supplementary Table S17. Comparison of observed and inverse probability weighted (IPW) outcome rates among patients classified as Phenotype 4.

Outcome	Unweighted rate	IPW-adjusted rate
Maternal metabolic or hypertensive complications	52.4%	51.1%
Delivery or neonatal compromise	32.4%	30.2%
Clinically actionable obstetric adverse event	57.1%	55.1%
Early preterm birth (<34 weeks)	10.8%	8.9%
Emergency cesarean section	27.0%	26.0%
1-minute Apgar score <7	8.1%	6.5%

Legend: IPW – inverse probability weighting.

Supplementary Table S18. Leakage-safe internal phenotype-replication performance and calibration of the Phenotype 4 translation models

Method	Target	Evaluation framework	AUROC (95% CI)	Sensitivity (95% CI)	Specificity (95% CI)	PPV (95% CI)	NPV (95% CI)	Brier score (95% CI)	Calibration intercept (95% CI)	Calibration slope (95% CI)
Leakage-safe cross-validated logistic surrogate model	Primary Phenotype 4 membership	Five-fold stratified OOF prediction; preprocessing and threshold selection performed within training folds	0.887 (0.823–0.940)	0.617 (0.491–0.737)	0.983 (0.976–0.989)	0.587 (0.467–0.705)	0.985 (0.978–0.991)	0.085 (0.077–0.093)	−3.418 (−3.967 to −3.001)	0.696 (0.586–0.857)
Simple points-based score (≥3)	Primary Phenotype 4 membership	Five-fold stratified held-out scoring; biomarker cutoffs derived within training folds; fixed ≥3 threshold applied to held-out scores	0.884 (0.828–0.931)	0.617 (0.491–0.732)	0.991 (0.986–0.995)	0.725 (0.595–0.839)	0.985 (0.979–0.990)	0.020 (0.014–0.026)	0.000 (−0.346 to 0.300)	0.966 (0.809–1.167)

Legend. AUROC – area under the receiver operating characteristic curve; OOF – out-of-fold; PPV – positive predictive value; NPV – negative predictive value; CI – confidence interval. The target for both models was membership in the primary AI-derived Phenotype 4 and not occurrence of a maternal, obstetric, delivery, or neonatal outcome. All performance estimates were obtained from held-out OOF predictions or scores. Ninety-five percent confidence intervals were calculated using 2,000 patient-level nonparametric bootstrap replicates. For the points-based score, AUROC was calculated from the raw OOF score, whereas the Brier score and calibration statistics were calculated from fold-specific score-to-probability mappings fitted exclusively within the training folds. The prevalence-only reference Brier score was 0.0365. The higher Brier score and non-ideal calibration parameters of the logistic surrogate indicate limited probability calibration despite good discrimination. These analyses quantify phenotype replication and should not be interpreted as clinical outcome prediction or external validation.

Supplementary Table S19. Complete specification of the simple points-based score for approximation of Phenotype 4 membership

Component	Cutoff or coding	Points	Basis of specification	Prespecified or data-derived
Maternal age	≥35 years	1	Conventional threshold for advanced maternal age	Prespecified clinical cutoff
BMI	≥30 kg/m^2	1	Conventional threshold for obesity	Prespecified clinical cutoff
Mean arterial pressure	≥90th percentile within the training fold	1	Upper-tail marker used to approximate the cardiometabolic profile of Phenotype 4	Data-derived percentile rule
Mean uterine artery PI	≥90th percentile within the training fold	1	Upper-tail first-trimester Doppler marker	Data-derived percentile rule
PAPP-A	≤10th percentile within the training fold	1	Lower-tail biochemical marker	Data-derived percentile rule
Chronic hypertension	Present	2	Cardiometabolic–vascular feature	Manually specified
Pre-existing diabetes	Present	1	Cardiometabolic feature	Manually specified
Previous preeclampsia	Present according to the normalized obstetric-history variable	2	Personal obstetric history of preeclampsia	Manually specified
**Total score range**	Sum of component points	**0–10**	—	—
**Classification threshold**	**≥3 points**	—	Threshold satisfying specificity ≥0.95 with the best sensitivity/PPV trade-off; independently selected in every training fold	Data-derived threshold

Legend. BMI – body mass index; PI – pulsatility index; PAPP-A – pregnancy-associated plasma protein A; PPV – positive predictive value. The score was constructed as an exploratory simplification of the AI-derived Phenotype 4 and was not designed to predict adverse pregnancy outcomes. Maternal age and BMI thresholds were prespecified clinically, whereas biomarker percentile thresholds were estimated independently within each training fold to prevent information leakage. All five training folds independently selected the same classification threshold of ≥3 points. Consequently, leakage-safe performance of the fixed ≥3 rule was evaluated in held-out folds without threshold re-optimization.

Supplementary Table S20. Specification of the logistic surrogate model used for Phenotype 4 translation

A. Predictor coding and preprocessing

Predictor	Model representation	Training-fold preprocessing	Missingness handling	Reference category
Maternal age	Continuous	Median imputation and z-standardization	Missingness indicator if retained	—
BMI	Continuous	Median imputation and z-standardization	Missingness indicator if retained	—
Mean arterial pressure (MAP)	Continuous	Median imputation and z-standardization	MAP missingness indicator	—
Mean uterine artery PI	Continuous	Median imputation and z-standardization	Missingness indicator if retained	—
PAPP-A	Continuous	Median imputation and z-standardization	PAPP-A missingness indicator	—
Chronic hypertension	Dummy-coded categorical	Training-fold encoding and column alignment	Missing category if applicable	No
Pre-existing diabetes	Dummy-coded categorical	Training-fold encoding and column alignment	Missing category if applicable	No diabetes
Maternal family history of preeclampsia	Dummy-coded categorical	Training-fold encoding and column alignment	Missing category if applicable	No
Conception method	Dummy-coded categorical	Training-fold encoding and column alignment	Missing category if applicable	IVF

B. Full-cohort descriptive logistic model

Predictor	β coefficient	Odds ratio
Intercept	−1.7525	0.173
Maternal age	0.2868	1.332
BMI	0.7827	2.187
Mean arterial pressure	1.0471	2.849
MAP missingness	0.3747	1.455
Mean uterine artery PI	−0.0448	0.956
PAPP-A	0.2045	1.227
PAPP-A missingness	−0.3876	0.679
Chronic hypertension	1.0941	2.986
Pre-existing diabetes, type 1	−0.1974	0.821
Pre-existing diabetes, type 2	−0.1983	0.820
Maternal family history of preeclampsia	0.0756	1.079
Ovulation-drug conception	−0.1131	0.893
Spontaneous conception	0.2848	1.329

The descriptive full-cohort model equation was:

logit{P(Phenotype 4)}=−1.7525+0.2868(age)+0.7827(BMI)+1.0471(MAP)+0.3747(MAP missing)−0.0448(UtA-PI)+0.2045(PAPP-A)−0.3876(PAPP-A missing)+1.0941(chronic hypertension)−0.1974(type 1 diabetes)−0.1983(type 2 diabetes)+0.0756(maternal family history of PE)−0.1131(ovulation-drug conception)+0.2848(spontaneous conception).\operatorname{logit}\{P(\text{Phenotype 4})\} = -1.7525 +0.2868(\text{age}) +0.7827(\text{BMI}) +1.0471(\text{MAP}) +0.3747(\text{MAP missing}) -0.0448(\text{UtA-PI}) +0.2045(\text{PAPP-A}) -0.3876(\text{PAPP-A missing}) +1.0941(\text{chronic hypertension}) -0.1974(\text{type 1 diabetes}) -0.1983(\text{type 2 diabetes}) +0.0756(\text{maternal family history of PE}) -0.1131(\text{ovulation-drug conception}) +0.2848(\text{spontaneous

conception}).$\text{logit}\{P(\text{Phenotype 4})\} = -1.7525 + 0.2868(\text{age}) + 0.7827(\text{BMI}) + 1.0471(\text{MAP}) + 0.3747(\text{MAP missing}) - 0.0448(\text{UtA-PI}) + 0.2045(\text{PAPP-A}) - 0.3876(\text{PAPP-A missing}) + 1.0941(\text{chronic hypertension}) - 0.1974(\text{type 1 diabetes}) - 0.1983(\text{type 2 diabetes}) + 0.0756(\text{maternal family history of PE}) - 0.1131(\text{ovulation-drug conception}) + 0.2848(\text{spontaneous conception})$.

Legend. BMI – body mass index; MAP – mean arterial pressure; UtA-PI – mean uterine artery pulsatility index; PAPP-A – pregnancy-associated plasma protein A; PE – preeclampsia. The logistic surrogate was fitted using L2-penalized logistic regression. Continuous predictors were imputed and standardized independently within each training fold; categorical variables were encoded using training-fold information, and held-out matrices were aligned to the training-fold design. The equation and coefficients shown above derive from a full-cohort descriptive fit and are provided only to specify the model structure. They were not used to estimate model performance. All reported performance estimates were obtained from held-out OOF predictions generated by five-fold stratified cross-validation.

Supplementary Table S21. Cross-validation structure and threshold selection for the Phenotype 4 translation analyses

Fold	Training patients, n	Training Phenotype 4, n	Held-out patients, n	Held-out Phenotype 4, n	Points-score threshold selected in training fold
1	1,266	48	317	12	≥3
2	1,266	48	317	12	≥3
3	1,266	48	317	12	≥3
4	1,267	48	316	12	≥3
5	1,267	48	316	12	≥3

Legend. Five-fold stratified cross-validation was generated once using random seed 20260708. Each held-out fold contained 12 patients assigned to primary Phenotype 4. For the logistic surrogate, median imputation, missingness handling, categorical encoding, standardization, model fitting, and probability-threshold determination were performed using training-fold data only. For the points-based score, the 90th-percentile MAP and uterine artery PI cutoffs and the 10th-percentile PAPP-A cutoff were recalculated within each training fold. The score threshold was also evaluated within the training data; all five folds independently selected ≥3 points. Final performance estimates were therefore based exclusively on held-out OOF predictions or scores and did not use apparent in-sample performance.

Supplementary Table S22. Incremental predictive performance of AI-derived phenotype membership.

Outcome	N	Events	Event rate (%)	Clinical AUROC	Clinical AUPRC	Clinical Brier score	Clinical + AI AUROC	Clinical + AI AUPRC	Clinical + AI Brier score	ΔAUROC	ΔAUPRC	ΔBrier
Clinically actionable obstetric adverse event	886	232	26.2	0.658	0.434	0.226	0.642	0.417	0.232	−0.016	−0.017	0.005
Delivery or neonatal compromise	832	134	16.1	0.558	0.213	0.243	0.555	0.208	0.244	−0.003	−0.005	0.001
Early preterm birth <34 weeks	824	27	3.3	0.614	0.104	0.201	0.698	0.161	0.192	**0.085**	0.058	−0.009
1-minute Apgar score <7	761	23	3.0	0.611	0.079	0.218	0.704	0.101	0.204	0.093	0.022	**−0.014**
Maternal metabolic or hypertensive complications	886	124	14.0	0.727	0.357	0.195	0.729	0.371	0.195	0.002	0.015	<0.001

Legend: AUROC, area under the receiver operating characteristic curve; AUPRC, area under the precision–recall curve; Δ, difference between the phenotype-augmented model and the baseline clinical model.

Supplementary Table S23. Bootstrap validation of incremental predictive performance after addition of AI-derived phenotype membership

Outcome	Metric	Estimate	95% CI	Bootstrap repetitions

Clinically actionable obstetric adverse event	ΔAUROC	−0.016	−0.034 to −0.001	1,000
	ΔAUPRC	−0.017	−0.043 to 0.007	1,000
	ΔBrier score	0.005	0.002 to 0.010	1,000
Delivery or neonatal compromise	ΔAUROC	−0.003	−0.033 to 0.027	1,000
	ΔAUPRC	−0.005	−0.025 to 0.014	1,000
	ΔBrier score	0.001	−0.003 to 0.005	1,000
Early preterm birth <34 weeks	ΔAUROC	**0.085**	**0.000 to 0.173**	1,000
	ΔAUPRC	0.058	−0.024 to 0.127	1,000
	ΔBrier score	−0.009	−0.019 to 0.001	1,000
1-minute Apgar score <7	ΔAUROC	0.093	−0.020 to 0.199	1,000
	ΔAUPRC	0.022	−0.062 to 0.096	1,000
	ΔBrier score	**−0.014**	**−0.026 to −0.002**	1,000
Maternal metabolic or hypertensive complications	ΔAUROC	0.002	−0.024 to 0.033	1,000
	ΔAUPRC	0.015	−0.027 to 0.059	1,000
	ΔBrier score	<0.001	−0.005 to 0.005	1,000

Legend: AUROC, area under the receiver operating characteristic curve; AUPRC, area under the precision–recall curve; CI, confidence interval; Δ, difference between the phenotype-augmented model and the baseline clinical model.
